# Comparison of Four Complete Chloroplast Genomes of Medicinal and Ornamental *Meconopsis* Species: Genome Organization and Species Discrimination

**DOI:** 10.1038/s41598-019-47008-8

**Published:** 2019-07-22

**Authors:** Xiaoxue Li, Wei Tan, Jiqi Sun, Junhua Du, Chenguang Zheng, Xiaoxuan Tian, Min Zheng, Beibei Xiang, Yong Wang

**Affiliations:** 1 College of Life Science, Nankai University, Weijin Road 94, 300071 Tianjin, China; 2 Tianjin State Key Laboratory of Modern Chinese Medicine, Tianjin University of Traditional Chinese Medicine, Poyang Lake Road 10, 301617 Tianjin, China; 3 College of Life and Geographic Sciences, Qinghai Normal University, 36 Wusixi Street, 810008 Qinghai, China; 4 School of Chinese Materia Medica, Tianjin University of Traditional Chinese Medicine, Poyang Lake Road 10, 301617 Tianjin, China

**Keywords:** Plant genetics, Medical genomics

## Abstract

High-throughput sequencing of chloroplast genomes has been used to gain insight into the evolutionary relationships of plant species. In this study, we sequenced the complete chloroplast genomes of four species in the *Meconopsis* genus: *M*. *racemosa*, *M*. *integrifolia* (Maxim.) Franch, *M*. *horridula* and *M*. *punicea*. These plants grow in the wild and are recognized as having important medicinal and ornamental applications. The sequencing results showed that the size of the *Meconopsis* chloroplast genome ranges from 151864 to 153816 bp. A total of 127 genes comprising 90 protein-coding genes, 37 tRNA genes and 8 rRNA genes were observed in all four chloroplast genomes. Comparative analysis of the four chloroplast genomes revealed five hotspot regions (*matK*, *rpoC2*, *petA*, *ndhF*, and *ycf1*), which could potentially be used as unique molecular markers for species identification. In addition, the *ycf1* gene may also be used as an effective molecular marker to distinguish Papaveraceae and determine the evolutionary relationships among plant species in the Papaveraceae family. Futhermore, these four genomes can provide valuable genetic information for other related studies.

## Introduction

The genus *Meconopsis* belongs to the Papaveraceae family of herb angiosperms and comprises approximately 49 species, 38 of which are found in China^1^. These plants are mainly distributed in the Himalayan foothills at an elevation of 2500–5500 m and are widely used in Tibetan folk medicine in China^2^. Detailed records of the medicinal usage of these plants have been written in the famous classic works on traditional Tibetan medicine, such as *Jingzhu Materia Medica*, *Yue Wang Yao Zhen*, and *Four Medical Codes*^3^. Recently, many kinds of isoquinoline alkaloids have been isolated from plants of the *Meconopsis* genus, and some have shown bioactivity, such as anti-inflammatory and analgesic activities^4^. Plants in this genus are also well known for their ornamental flowers and are widely used in horticultural gardening, with names such as *fairy grass* and *Himalayan poppy*. These plants are iconic in Tibet and Yunnan and play a significant role in the local Tibetan economy, as they are among the top ten ornamental flowering plants in the region^2^. Howere, overexploitation and anthropogenic habitat destruction are increasingly threatening the survival of many wild *Meconopsis* species. *Meconopsis punicea* has been listed as an endangered species on the China Species Red List^5^.

To understand the evolutionary relationships of plant species in the *Meconopsis* genus and in the Papaveraceae family, it is important to obtain genetic information or molecular markers of individual species. This “barcode” can also aid in medicinal usage, for which the accurate identification of species is required, as the regions and sources of species are often complex or unknown^6–8^ and can affect the efficacy of the final medicinal product.

Recent chloroplast genomic research has provided large quantities of data that are useful for selecting pertinent markers to resolve obscure phylogenetic relationships in seed plants^9^. At present, nearly 3000 complete chloroplast genomes are available in the NCBI database (https://www.ncbi.nlm.nih.gov/genomes/GenomesGroup.cgi?taxid=2759&opt=plastid)^10^. However, there is only one sequence from the chloroplast DNA of *Meconopsis* species in GenBank^11^.

In this study, we sequenced and assembled the chloroplast genomes of four *Meconopsis* species using a next-generation sequencing platform. We report the assembly, annotation and analysis of the chloroplast genomes of *Meconopsis racemosa*, *Meconopsis integrifolia* (Maxim.) Franch, *Meconopsis horridula* and *Meconopsis punicea*. We also constructed phylogenetic trees to perform comparisons among chloroplast genomes published for other plant species in related families. This study expands our understanding of the diversity of chloroplast genomes of *Meconopsis* species and their evolutionary relationships and provides fundamental data for the genetic engineering of *Meconopsis* chloroplasts.

## Results and Discussion

### Chloroplast genome sequencing, assembly and validation

**Figure 1:**
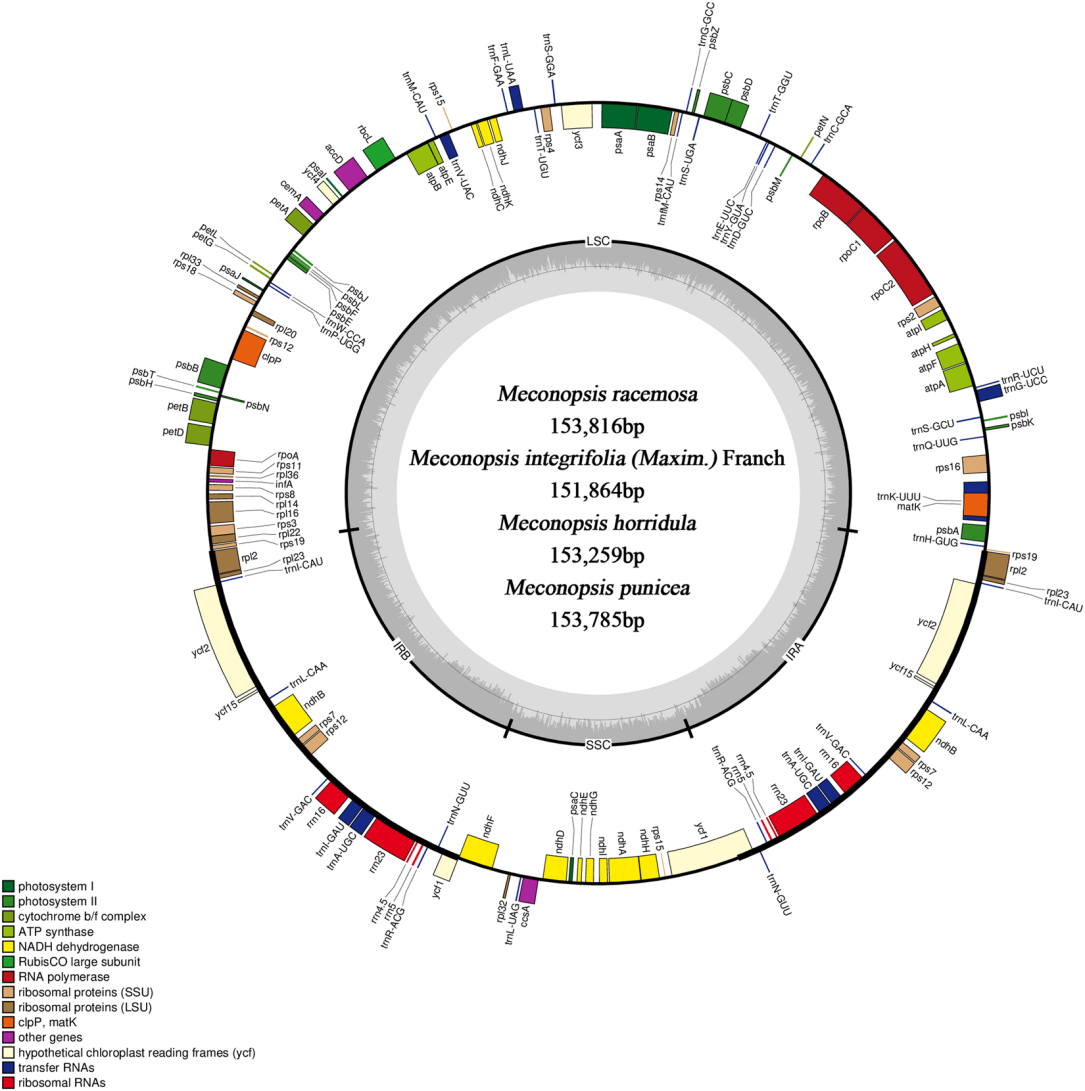
Chloroplast genome maps of *M*. *racemosa*, *M*. *integrifolia* (Maxim.) Franch, *M*. *horridula* and *M*. *punicea*. Genes inside circles are transcribed clockwise, genes outside circles are transcribed counterclockwise. The light gray inner circle corresponds to the AT content, and the dark gray circle corresponds to the GC content. Genes belonging to different functional groups are shown in different colors.

Using the Illumina HiSeq 2000 system, we sequenced the complete chloroplast genomes of four *Meconopsis* species, *M*. *racemosa*, *M*. *integrifolia* (Maxim.) Franch, *M*. *horridula* and *M*. *punicea*. Raw data were generated with an average read length of 150 bp. The complete sequences of the four chloroplast genomes were assembled by both de novo and reference-based assembly. Gaps were validated using PCR-based sequencing with one primer pair (Supplementary Table 1). The final high-quality chloroplast genome sequences were submitted to GenBank (Accession Numbers: *M*. *racemosa*, MK533649; *M*. *integrifolia* (Maxim.) Franch, MK533647; *M*. *horridula*, MK533646; *M*. *punicea*, MK533648), and the corresponding genome maps are shown in Fig. 1.

### Chloroplast genome structural features and gene content

It was previously reported that the chloroplast genomes of angiosperms are conserved in their genomic structure in terms of gene number and order, although IR expansion or contraction occur frequently^12,13^. The *Meconopsis* chloroplast genomes are in accordance with this observation, and their genome structures are similar to those of other Papaveraceae species^14^. All of the *Meconopsis* chloroplast genomes display the typical quadripartite structure of angiosperm cpDNA, which consists of a pair of IR regions (51306–51988 bp) separated by an LSC region (82809–83982 bp) and an SSC region (17729–17898 bp). These four chloroplast genomes are highly conserved in gene content, gene order, and intron number. The *Meconopsis* chloroplast genomes harbor 127 genes, 90 coding proteins, 37 coding tRNAs and 8 coding rRNAs. Some genes are duplicated in the IR region, among which ten are protein-coding genes (*rpl2*, *rpl12*, *rps12*, *rps15*, *rps16*, *rps19*, *ndhB*, *ycf1*, *ycf15* and *ycf2*), four are ribosomal RNA genes (rrn4.5, rrn5, rrn16, rrn23) and six are transfer RNA genes (trnL-CAA, trnN-GUU, trnR-ACG, trnA-UGC, trnI-GAU and trnV-GAC) (Table 1). Fifteen protein-coding genes (*petB*, *petD*, *ndhA*, *ndhB*, *atpF*, *rps12*, *rps15*, *rps16*, *rps19*, *rpl2*, *rpl12*, *rpl16*, *rpoC1*, *clpP*, and *ycf3*) contain one or more introns. The A content ranged from 30.4 to 30.5%, the C content ranged from 19.7 to 19.8%, the G content ranged from 18.8 to 19%, the T content ranged from 30.8 to 31%, and the GC content ranged from 38.5 to 38.8%, indicating nearly identical levels among the four *Meconopsis* chloroplast genomes (Table 2).

**Table 1:** Summary of assembly data for the *Meconopsis* chloroplast genome.

Species	*Meconopsis racemosa*	*Meconopsis integrifolia* (*Maxim*.) Franch	*Meconopsis horridula*	*Meconopsis punicea*
Genome size (bp)	153816	151864	153785	153259
IR (bp)	51988	51306	51988	51548
LSC (bp)	83930	82809	83899	83982
SSC (bp)	17898	17749	17898	17729
Total number of genes	127	127	127	127
rRNA	8	8	8	8
tRNA	37	37	37	37
Protein-coding genes	90	90	90	90
A %	30.4	30.4	30.4	30.5
C %	19.8	19.8	19.8	19.7
G %	18.9	19	18.9	18.8
T %	30.9	30.8	30.9	31
G C%	38.7	38.8	38.8	38.5

**Table 2:** Chloroplast genome gene content and functional classification in *M*.

Category	Group	Genes
Self-replication	Large subunit of ribosome (LSU)	*rpl14*, *rpl16*^a^, *rpl2*^a,b^, *rpl2*^a,b^, *rpl20*, *rpl22*, *rpl23*^b^, *rpl23*^b^, *rpl32*, *rpl33*, *rpl36*
	Small subunit of ribosome (SSU)	*rps11*, *rps12*^a,b^, *rps14*, *rps15*^a,b^, *rps16*^a^, *rps18*, *rps19*^a,b^, *rps2*, *rps3*, *rps4*, *rps7*^b^, *rps8*
	DNA dependent RNA polymerase	*rpoA*, *rpoB*, *rpoC1*^a^, *rpoC2*
	Ribosome RNA	*rrn16*^b^, *rrn23*^b^, *rrn4*.*5*^b^, *rrn5*^b^
	Transfer RNAs (tRNA)	*trnC*-*GCA*, *trnD*-*GUC*, *trnE*-*UUC*, *trnF*-*GAA*, *trnfM*-*CAU*, *trnG*-*GCC*, *trnH*-*GUG*, *trnI*-*CAU*, *trnI*-*CAU*, *trnL*-*CAA*^b^, *trnL*-*UAG*, *trnM*-*CAU*, *trnN*-*GUU*^b^, *trnP*-*UGG*, *trnQ*-*UUG*, *trnR*-*ACG*^b^, *trnR*-*UCU*, *trnS*-*GCU*, *trnS*-*GGA*, *trnS*-*UGA*, *trnT*-*GGU*, *trnT*-*UGU*, *trnV*-*GAC*^b^, *trnW*-*CCA*, *trnY*-*GUA* *trnK*-*UUU*^a^, *trnG*-*UCC*^a^, *trnV*-*UAC*^a^, *trnA*-*UGC*^a^, *trnL*-*UAA*^a^, *trnI*-*GAU*^a^
Photosynthesis	Photosystem I	*psaA*, *psaB*, *psaC*, *psaI*, *psaJ*
	Photosystem II	*psbA*, *psbB*, *psbC*, *psbD*, *psbE*, *psbF*, *psbH*, *psbI*, *psbJ*, *psbK*, *psbL*, *psbM*, *psbN*, *psbT*, *psbZ*
	NADH dehydrogenase	*ndhA*^a^, *ndhB*^a,b^, *ndhC*, *ndhD*, *ndhE*, *ndhF*, *ndhG*, *ndhH*, *ndhI*, *ndhJ*, *ndhK*
	Cytochrome b/f complex	*petA*, *petB*^a^, *petD*^a^, *petG*, *petL*, *petN*
	Subunits of ATP synthase	*atpA*, *atpB*, *atpE*, *atpF*^a^, *atpH*, *atpI*
	Large subunit of rubisco	*rbcL*
Other genes	Translational initiation factor	*infA*
	ATP-dependent protease subunit p gene	*clpP* ^a^
	Maturase	*matK*
	Envelop membrane protein	*cemA*
Unknown function	Subunit of acetyl-CoA-carboxylase	*accD*
	C-type cytochrome synthesis gene	*ccsA*
	Hypothetical chloroplast reading frames	*ycf1*^b^, *ycf15*^b^, *ycf2*^b^, *ycf3*^a^, *ycf4*

*racemosa*, *M*. *integrifolia* (Maxim.) Franch, *M*. *horridula* and *M*. *punicea*. ^a^Genes containing introns; ^b^Two gene copies in IR.

### Amino acid abundance and codon usage

Codon usage plays an important role in shaping chloroplast genome evolution. Mutational bias has been reported to have an essential role in this process^15^. As shown in Supplementary Tables 2–5, the 90 protein-coding genes are encoded by 26338, 26365, 26342 and 26337 codons in the chloroplast genomes of *M*. *racemosa*, *M*. *integrifolia* (Maxim.) Franch, *M*. *horridula* and *M*. *punicea*, respectively. Leucine (11.1–9.5%) was the most abundant amino acid among the proteins encoded by the chloroplast genes. Cysteine (1.2–1.7%) was the least abundant amino acid in the proteins encoded by chloroplast genes in the *M*. *racemosa*, *M*. *integrifolia* (Maxim.) Franch, *M*. *horridula* and *M*. *punicea* chloroplast genomes. Leucine and isoleucine are the most commonly observed amino acids in the proteins of chloroplast genomes of angioperms^16^.

**Figure 2:**
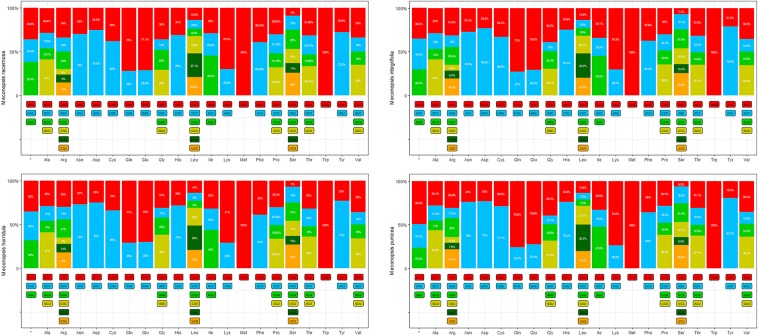
Codon content of twenty amino acids and stop codons in all protein-coding genes of the chloroplast genomes of four *Meconopsis* species.

We calculated and summarized the codon usage of the chloroplast genomes in these four plants (Fig. 2). The codon UUA, for leucine, occurred at the highest proportion in all four species (27.1–30.3%). There were a total of 711 codons encoding tRNA genes in the *M*. *racemosa*, *M*. *integrifolia* (Maxim.) Franch and *M*. *horridula* chloroplast genomes, but only 704 codons in the tRNA-encoding genes in *M*. *punicea* (Supplementary Tables 2–5), indicating that codons ending in U and A were common; perhaps the variation in the tRNA-encoding genes is related to species evolution.

We also calculated the relative synonymous codon usage (RSCU) in the chloroplast genomes of the four species. Usage of the start codon methionine AUG and tryptophan UGG had no bias (RSCU = 1). All preferred relative synonymous codons (RSCU >1) ended with an A or a U, except for UUG (all 4 species), UCC (*M*. *integrifolia* (Maxim.) Franch, *M*. *horridula* and *M*. *punicea*) and UAG (*M*. *integrifolia* (Maxim.) Franch and *M*. *punicea*) (Supplementary Tables 2–5).

### Plastid RNA editing prediction

RNA editing is a generic term comprising a variety of processes that alter the DNA-encoded sequence of a transcribed RNA by inserting, deleting or modifying nucleotides in a transcript^17^. Chloroplast RNA editing was first discovered in 1991. Nearly 30 years after the discovery of C-to-U editing in plant chloroplasts, the field has recently expanded tremendously in several research directions^18^. RNA editing provides a way to create transcript and protein diversity^19^. In higher plants, some chloroplast RNA editing sites are conserved^20^.

To gain insight into the RNA editing sites in *Meconopsis* plants, we predicted 92, 78, 84 and 94 RNA editing sites out of 27, 26, 28 and 28 plastid genes in the chloroplast genomes of *M*. *racemosa*, *M*. *integrifolia* (Maxim.) Franch, *M*. *horridula* and *M*. *punicea*, respectively, with PREP (Supplementary Tables 6–9). In these four species, the amino acid conversion from S to L was the most frequent type of conversion. As previously reported, with increased amino acids, the conversion from S to L becomes more frequent^21^. This finding indicated that the evolutionary conservation of RNA editing is essential^22,23^.

### Simple sequence repeats and repetitive sequence analysis

Tandem repeat sequences consisting of 1–6 nucleotide repeat units are known as simple sequence repeats (SSRs), or microsatellites^24^. SSRs are valuable molecular markers with a high degree of variation within species and have been used in many population genetics and polymorphism investigations. Using the MISA software tool, we analyzed the occurrences and types of SSRs in the four *Meconopsis* chloroplast genomes. These genomes all have SSRs, and the majority of which are mono- and dinucleotide repeats, which were identified 88 and 29 times, respectively. The mononucleotide repeats were A/T repeats, and 82.8% of the dinucleotide repeats were AT/AT repeats (Table 3). Although the AT richness in the SSRs of the four chloroplast genomes of *Meconopsis* species was similar to that identified in previous studies, which suggested that SSRs found in the chloroplast genome are generally composed of polythymine (T) or polyadenine (A) repeats^25^, the number of SSRs differs among the different species (40 in *M*. *racemosa*, 33 in *M*. *integrifolia* (Maxim.) Franch, 38 in *M*. *horridula* and 34 in *M*. *punicea*; Table 3). These findings indicate that SSRs can be used as molecular markers to identify these plant species.

**Table 3:** Types and numbers of SSRs in the chloroplast genomes of *M*. *racemosa*, *M*. *integrifolia* (Maxim.) Franch, *M*. *horridula* and *M*. *punicea*.

SSR type	Repeat unit	Species			
		*Meconopsis racemosa*	*Meconopsis integrifolia* (Maxim.) Franch	*Meconopsis horridula*	*Meconopsis punicea*
Mono	A/T	24	22	23	19
Di	AG/CT	1	1	1	1
	AC/GT	0	0	0	1
	AT/AT	7	4	7	6
Tri	AAT/ATT	2	2	2	2
Tetra	AAAT/ATTT	3	2	3	2
	AACC/GGTT	1	1	1	1
	AGAT/ATCT	1	1	1	0
	ATCC/ATGG	0	0	0	1
Hexa	AATGAT/ATCATT	0	0	0	1
	AAAAT/ATTTT	1	0	0	0

**Figure 3:**
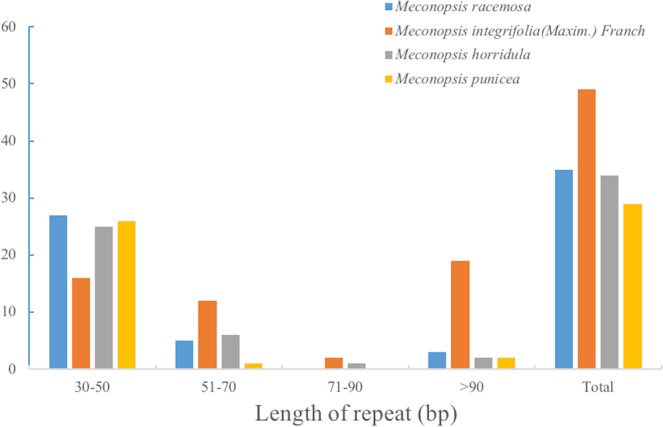
Frequency of repeat sequences of the *M*. *racemosa*, *M*. *integrifolia* (Maxim.) Franch, *M*. *horridula* and *M*. *punicea* chloroplast genomes determined by REPuter.

More complex and longer repeat sequences may play an important roles in sequence divergence and genomes^26^. In these four *Meconopsis* chloroplast genomes, we found that the length of repeated sequences ranged mainly from 30 to 90 bp, similar to the lengths reported in other angiosperm plants^25,27,28^. The numbers of repeats with at least 30 base pairs (bp) per repeat unit in the *M*. *racemosa*, *M*. *integrifolia* (Maxim.) Franch, *M*. *horridula*, and *M*. *punicea* chloroplast genomes are 35, 49, 34 and 29, respectively. The *M*. *racemosa* chloroplast genome contains 27 repeats of 30–50 bp, 5 repeats of 51–70 bp, and 3 repeats longer than 90 bp. The *M*. *integrifolia* (Maxim.) Franch chloroplast genome contains 16 repeats of 30–50 bp, 12 repeats of 51–70 bp, 2 repeats of 71–90 bp and 19 repeats longer than 90 bp. The *M*. *horridula* chloroplast genome contains 25 repeats of 30–50 bp, 6 repeats of 51–70 bp, 1 repeat of 71–90 bp and 2 repeats longer than 90 bp. The *M*. *punicea* chloroplast genome contains 26 repeats of 30–50 bp, 1 repeat of 51–70 bp, and 2 repeats longer than 90 bp (Fig. 3).

### Divergent hotspots in the *Meconopsis* chloroplast genome

Molecular markers with nucleotide diversity over 1.5% have been reported as highly variable regions that can be used for phylogenetic analysis and species identification in seed plants^29,30^. Currently, there are few molecular biology-based studies of *Meconopsis* plants, and there is no uniform molecular marker for species identification^31–35^.

**Figure 4:**
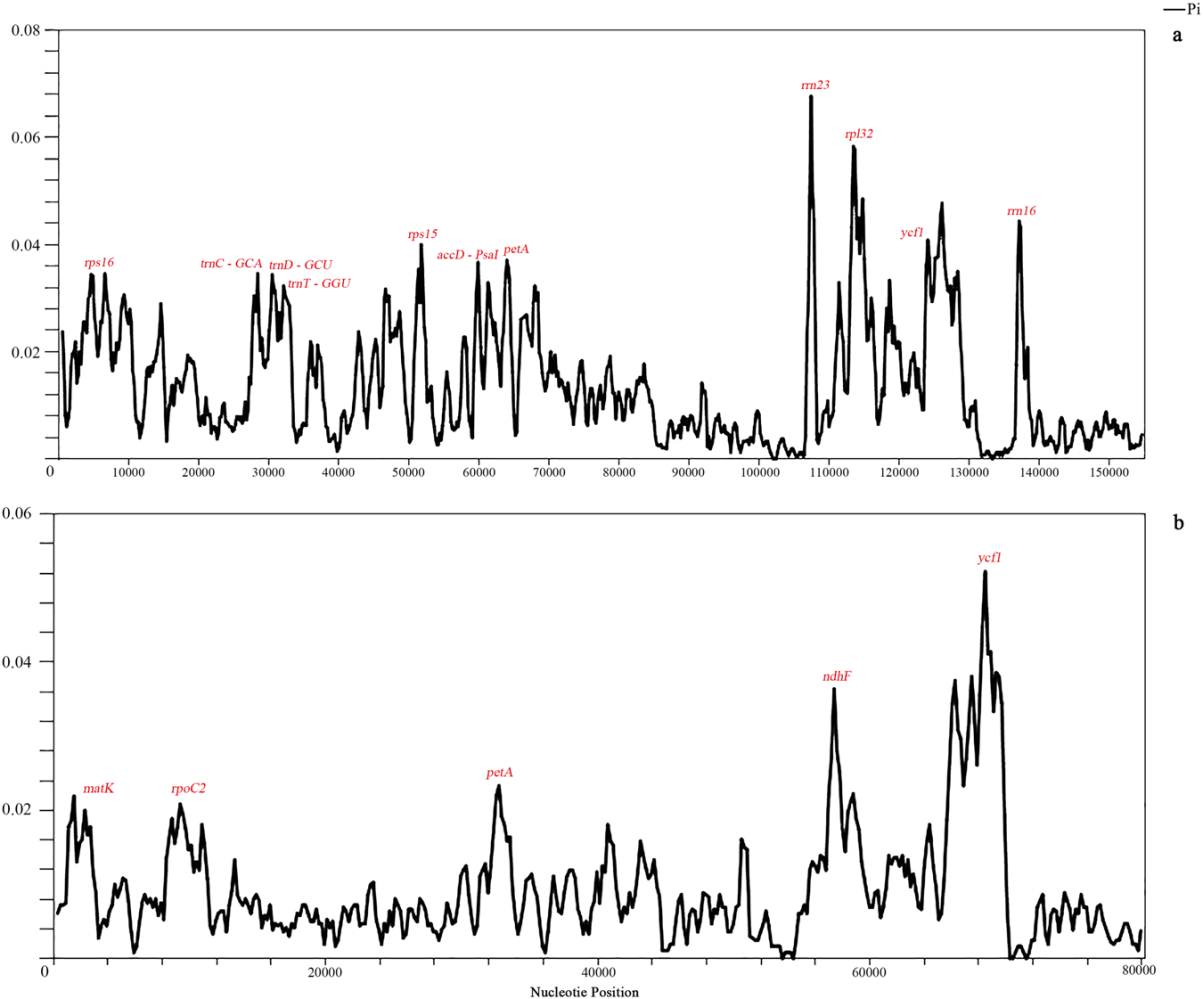
Nucleotide variability (%) values between pairs of the four *Meconopsis* species. (**a**) Using four species whole genomes; (**b**) Using four species coding regions.

A SNP (single nucleotide polymorphism) marker is a single base change in a DNA sequence, typically with two possible nucleotide alternatives at a given position^36^. A total of 176, 2459, 36, 2982 SNPs were found in *M*. *racemosa*, *M*. *integrifolia* (Maxim.) Franch, *M*. *horridula* and *M*. *punicea*, respectively. To reveal the sequence divergence levels, the nucleotide variability values within 800 bp in all four chloroplast genomes were calculated with DnaSP 6.10.03 software. The values ranged from 0 to 0.07, revealing slight differences among the genomes. For example, the *p*-distance between *M*. *racemosa* and each of *M*. *integrifolia* (Maxim.) Franch, *M*. *horridula* and *M*. *punicea* is 0.016, 0.001 and 0.018, respectively. These divergence hotspot regions can provide information for marker development for phylogenetic analyses of *Meconopsis* species. Overall, the results reveal higher divergence in noncoding regions than in coding regions. Using whole chloroplast genomes, we found that some regions differ among the four species, such as *rps16*, *trnC*-*GCA*, *trnD*-*GCU*, *trnT*-*GGU*, *rps15*, *accD*-*PsaI* and *petA* (Fig. 4a). The coding regions with marked differences include the *matK*, *rpoC2*, *petA*, *ndhF* and *ycf* genes (Fig. 4b). These genes could be utilized as potential phylogenetic markers to reconstruct the phylogeny in this genus. Qu Yan *et al*. reported that the *ndhF* gene could not be used to distinguish *M*. *racemosa* from *M*. *horridula*^37^. However, our present study shows that the sequence of the *ndhF* gene in the chloroplast genome differs between these two species is distinct.

Divergent hotspots of chloroplast genomes have been used to identify species in other plants of the Papaveraceae family. Jianguo Zhou *et al*. used *ycf1*, *rpoB*-*trnC*, *trnD*-*trnT*, *petA*-*psbJ*, *psbE*-*petL* and *ccsA*-*ndhD* sequences in the chloroplast genome to distinguish *Papaver orientale* and *Papaver rhoeas*^14^. Zhe Zhang *et al*.^38^ analyzed the phylogeny of 15 species from the Papaveraceae family based on the nuclear gene ITS sequence, the chloroplast gene *rbcL* sequence, and the combined sequences of these genes.

### Comparisons of the chloroplast genomes among nine species in the Papaveraceae family

We compared the 9 known chloroplast genome sequences of species in the Papaveraceae family (*M*. *racemosa*, *M*. *integrifolia* (Maxim.) Franch, *M*. *horridula*, *M*. *punicea*, *Macleaya microcarpa* (MH394383.1), *Coreanomecon hylomeconoides* (KT274030.1), *Papaver somniferum* (KU204905.1), *Papaver rhoeas* (MF943221.1) and *Papaver orientale* (MF943222.1)). The results indicated that species with the largest chloroplast genome is the *M*. *microcarpa* (161118 bp) and that with the smallest is *M*. *integrifolia* (Maxim.) Franch genome (151864 bp) (Table 1). The *M*. *microcarpa* (161118 bp) genome was used as the reference genome.

**Figure 5:**
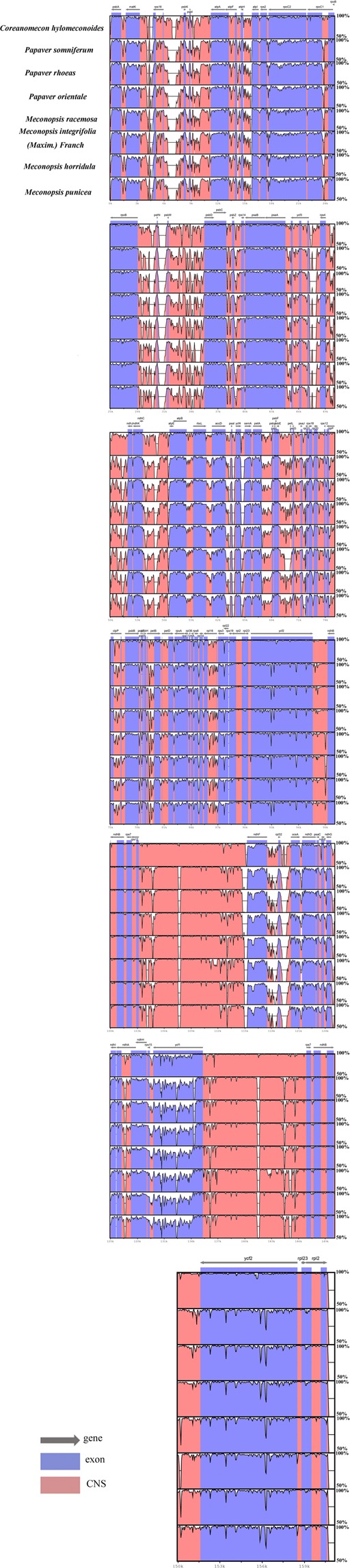
Sequence identity plot comparing the eight chloroplast genomes with *Macleaya microcarpa* as a reference by using mVista. Pink bars represent noncoding sequences (CNS), and white peaks represent genomic differences. The y-axis represents the percentage identity (shown: 50–100%).

Next, we used the online program mVISTA to analyze gene order and content in the chloroplast genome. We found that the gene order and contents of the *Meconopsis* plants are similar to those of other members of the Papaveraceae family (Fig. 5). Similar to other plant species, all *Meconopsis* species have conserved chloroplast genomes, their coding regions are more conserved than their noncoding regions, and their IR regions are more conserved than their LSC and SSC regions^16,39,40^.

### Altitude and plant distribution

Altitude influences ecological factors such as water and temperature, which affects plant genetic variation and population differentiation^41^. In this study, the plant materials of *M*. *racemosa* and *M*. *integrifolia* (Maxim.) Franch were mainly collected from the Bayan Har mountains, Qinghai Province. This region has a cold continental climate with an average altitude of over 5000 m. The plant materials of *M*. *horridula* were collected from Matuo Country, Guoluo Tibetan Autonomous Prefecture, Qinghai Province. This region has an alpine grassland climate with an average annual temperature of −4 °C and an average altitude of over 4000 m. The plant materials of *M*. *punicea* were mainly collected in Chindu Country, Qinghai Province. This region has an average altitude of over 4000 m. Studies have shown that the evolutionary relationships of plants are affected by altitude^42,43^. The plant materials used in this study were collected in the same area but at different altitudes: *M*. *racemosa* 4232 m; *M*. *integrifolia* (Maxim.) Franch, 4695 m; *M*. *horridula*, 4289 m; and *M*. *punicea*, 4639 m. According to traditional plant morphology taxonomy, *M*. *racemosa* is more closely related to *M*. *horridula* than to other *Meconopsis* species and is more distantly related to *M*. *integrifolia* (Maxim.) Franch and *M*. *punicea*^44^, which is consistent with both the phylogenetic results of this study and the altitudes of their distributions. Although they are distributed in the same region, there is evident genetic isolation among them. We speculate that altitude may be an important ecological factor that affects the evolution of *Meconopsis* plants.

### Phylogenetic analysis

**Figure 6:**
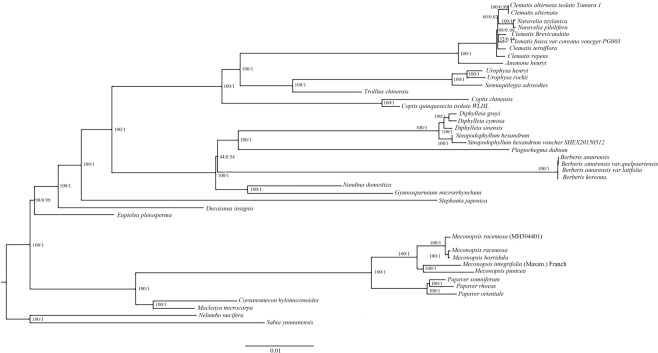
Phylogenetic tree reconstruction of the 42 species inferred from Bayesian inference (BI) and maximum likelihood (ML) based on 90 protein-coding genes. Numbers above the lines represent BI/ML posterior probabilities.

With improvements and advancements in techniques, increasing numbers of chloroplast genome sequences have been used to reconstruct plant phylogenies^45^. To identify the phylogenetic positions of *M*. *racemosa*, *M*. *integrifolia* (Maxim.) Franch, *M*. *horridula* and *M*. *punicea* within the *Meconopsis* genus, Bayesian inference (BI) and maximum likelihood (ML) methods of phylogenetic analysis were performed based on 90 protein-coding gene datasets from 40 plant taxa, with *Sabia yunnanensis* and *Nelumbo nucifera* used as outgroups. Both the BI and ML trees have similar phylogenetic topologies, and most nodal support values were high (Fig. 6). Using this reconstructions, *M*. *racemosa*, *M*. *racemosa* (MH394401)^11^ and *M*. *horridula* were grouped together, as were *M*. *integrifolia* (Maxim.) Franch and *M*. *punicea*. These species are closely related to the *Papaver* genus within the Papaveraceae family.

In addition, we found that *M*. *racemosa*, *M*. *horridula* and *M*. *racemosa* (MH394401)^11^ were grouped together. For several years, the delimitation of *M*. *racemosa* and *M*. *horridula* in the genus has been highly controversial^46^. Fedd, Kingdon-Ward and Prain *et al*. considered *M*. *racemosa* and *M*. *horridula* to be the same species^46^. However, in *Tibetan Flora*, *M*. *racemosa* is described as a variant of *M*. *horridula*. *M*. *racemosa* and *M*. *racemosa* (MH394401)^11^ were distributed on different branches but are the same species. Incomplete lineage sorting, insufficient informative characters, hybridization or plastid capture could be responsible for the incongruent phylogenetic positions of this species^47,48^.

**Figure 7:**
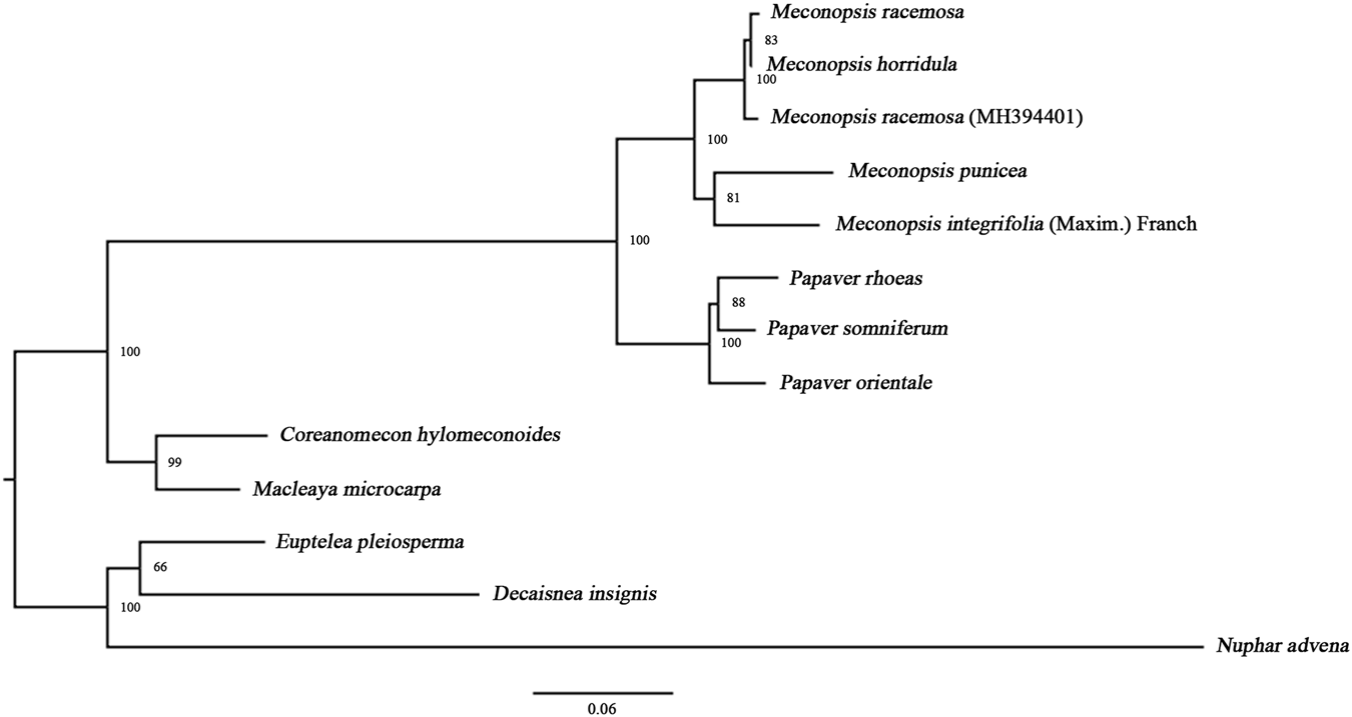
Phylogenetic tree reconstruction of the 13 species inferred from maximum likelihood (ML), based on the *ycf1* genes.

We used the five gene markers (*matK*, *rpoC2*, *petA*, *ndhF* and *ycf1* genes), screened by divergent hotspots in the *Meconopsis* chloroplast genomes, to construct five phylogenetic trees of these four *Meconopsis* plants and five other plants from the Papaveraceae family (*P*. *somniferum*, *P*. *rhoeas*, *P*. *orientale*, *Macleaya microcarpa* and *Coreanomecon hylomeconoides*) using *Decaisnea insignis*, *Euptelea pleiosperma* and *Nuphar advena* as outgroups (Fig. 7 and Supplementary Figs 1–4). The results showed that *M*. *racemosa*, *M*. *racemosa* (MH394401)^11^ and *M*. *horridula* are grouped together and that *M*. *integrifolia* (Maxim.) Franch and *M*. *punicea* are grouped together. Among the five genes, the *rpoC2* gene is not a suitable for potential DNA barcoding of *Meconopsis* plants, and the *ycf1* gene has the highest node support value in the phylogenetic tree, which is consistent with previous reports that have used *ycf1* to distinguish unknown Papaveraceae plants^14,49^. In *Tibetan Flora*, *M*. *racemosa* is described as a variant of *M*. *horridula* on account of the similar morphological characterization of these taxa and the consistent ITS sequence. However, Dou *et al*.^35^, using the ITS2 sequence, and Ni *et al*.^34^, using the *psbA*-*trnH* sequence, constructed an evolutionary trees and found that these taxa clustered in different branches.

The chloroplast genome usually contains uniparentally inherited DNA, which is well suited for studying the evolutionary history of plants, such as dating a common ancestor^50^. Yuan *et al*. used the chloroplast genome sequence of *trnL*-*trnF* and found that *M*. *punicea* is the mother of the hybrid species *Meconopsis* × *cookei* (Papaveraceae) and that *M*. *quintuplinervia* is the father^33^.

## Conclusions

In this study, we used the Illumina HiSeq 2000 system to sequence the complete chloroplast genomes of four *Meconopsis* species: *M*. *racemosa*, *M*. *integrifolia* (Maxim.) Franch, *M*. *horridula* and *M*. *punicea*. We demonstrate that these four *Meconopsis* species are divided into two groups, with *M*. *racemosa* and *M*. *horridula* in one group and *M*. *integrifolia* (Maxim.) Franch and *M*. *punicea* in the other. By comparing the chloroplast genome sequences, we were able to retrieve all genetic resources, including SNPs, SSRs, repetitive sequence, codon usage, RNA editing prediction, ‘hotspot’ regions and phylogenomic analysis. These resources will provide chloroplast genome molecular markers for the identification of these *Meconopsis* species. We also used four hotspot genes (*matK*, *petA*, *ndhF* and *ycf1*) to construct phylogenetic trees and clearly distinguish these species.

With the development of plant science, plastid transformation is becoming an important tool. The limited availability of complete chloroplast genomic information is one of the major factors preventing the extension of this technology to valuable plants. The *Meconopsis* chloroplast genome data obtained in this study could be applied in biotechnology and provide useful information for designing transformation vectors in the future.

## Materials and Methods

### Plant material and DNA extraction

The plant materials used in this study were seeds collected from *M*. *racemosa*, *M*. *integrifolia* (Maxim.) Franch, *M*. *horridula* and *M*. *punicea* in Qinghai Province. All samples were identified by Professor Junhua Du, who is affiliated with Qinghai Normal University. Total genomic DNA was isolated from seeds using the Mag-MK Plant Genomic DNA extraction kit (Sangon Biotech, Shanghai, China), and DNA quality was assessed based on spectrophotometry and electrophoresis in a 1% (w/v) agarose gel. Total DNA samples were chosen for Illumina 2000 sequencing.

### Chloroplast genome assemblage and annotation

For these four species, the high-throughput sequencing data were qualitatively assessed and assembled using NOVOPlasty 2.6.3. Gaps in the cpDNA sequences were filled by PCR amplification and Sanger sequencing. The annotations of the chloroplast genomes were performed with Geneious 8.0.4, DOGMA^51^, CPGAVAS^52^ and CPGAVAS2^53^ followed by manual correction. The tRNAs were verified by the online tRNAscan-SE 1.21 search server. All the annotations were manually checked against the references (NC_029434.1 and NC_031446.1). The genome maps were drawn by OGDRAW. The entire chloroplast genome sequences of *M*.*racemosa*, *M*. *integrifolia* (Maxim.) Franch, *M*. *horridula* and *M*. *punicea*, along with the gene annotations, were submitted to GenBank (Accession Numbers: *M*. *racemosa*, MK533649; *M*. *integrifolia* (Maxim.) Franch, MK533647; *M*. *horridula*, MK533646; *M*. *punicea*, MK533648).

### Codon usage

Codon usage was determined for all protein-coding genes. The relative synonymous codon usage (RSCU) values and codon usage were determined with MEGA7, which was used to reveal the characteristics of the variation in synonymous codon usage^54^.

### Simple sequence repeats and repetitive sequence analysis

Chloroplast microsatellites were identified in a high-quality sequence of clusterbean by using the MISA Perl script^55^. The minimum numbers for the SSR motifs were 10, 5, 4, 3, 3 and 3 for mono-,di-,tri-,tetra-,penta-,and hexanucleotide repeats, respectively. REPuter was used to identify forward repeats, reserve sequences, complementary and palindromic sequences, with a minimum repeat size of 30 bp and 90% sequence identity^56^.

### Prediction of RNA editing sites

Twenty-eight protein-coding genes of *M*. *racemosa*, *M*. *integrifolia* (Maxim.) Franch, *M*. *horridula* and *M*. *punicea* were used to predict potential RNA editing sites using the Predictive RNA Editor for Plants (PERP) suite (http://prep.unl.edu) with a cutoff value of 0.8.

### Genome comparison

MAFFT was used to align the chloroplast genomes^57^. The complete chloroplast genomes of *M*. *racemosa*, *M*. *integrifolia* (Maxim.) Franch, *M*. *horridula* and *M*. *punicea* were compared using mVISTA^58^.

### Divergent hotspots identification

The *M*. *racemosa*, *M*. *integrifolia*(Maxim.) Franch, *M*. *horridula* and *M*.*punicea* chloroplast genome sequences were aligned using MAFFT and were manually adjusted using Geneious 8.0.4. To analyze nucleotide diversity, we conducted a sliding window analysis using DnaSP version 6.10.03. software^59^. The window length was set to 800 bp, and the step size was 200 bp.

### Phylogenetic analysis

The chloroplast genome sequences of *M*. *racemosa*, *M*. *integrifolia*(Maxim.) Franch, *M*. *horridula*, *M*. *punicea* and those of 38 other species were collected from NCBI (Supplementary Table 10) were used for phylogenetic analysis. All of the coding sequences from the 42 species were aligned with the MAFFT method based on codons by Geneious 8.0.4. The best nucleotide substitution model (GTR + G + I) was tested, and a maximum likelihood (ML) tree (1000 bootstrap replicates) was constructed with RAxML software^60^. BI analyses were conducted using GPU MrBayes. The GTR + I + G substitution model was used for BI. In the BI analyses, two simultaneous runs of 10000000 generations were conducted for the matrix. Each set was sampled every 1000 generations with a burn-in of 25%. The *matK*, *rpoC2*, *petA*, *ndhF* and *ycf1* gene sequences of *M*. *racemosa*, *M*. *integrifolia*(Maxim.) Franch, *M*. *horridula*, *M*. *punicea* and 9 other species were collected from NCBI. Maximum likelihood (ML) analyses were conducted using RAxML software with the GTR model^61^.

## Supplementary information

Supplementary
