# Author Correction: Comparison of Four Complete Chloroplast Genomes of Medicinal and Ornamental *Meconopsis* Species: Genome Organization and Species Discrimination

**DOI:** 10.1038/s41598-019-51738-0

**Published:** 2019-10-17

**Authors:** Xiaoxue Li, Wei Tan, Jiqi Sun, Junhua Du, Chenguang Zheng, Xiaoxuan Tian, Min Zheng, Beibei Xiang, Yong Wang

**Affiliations:** 1 College of Life Science, Nankai University, Weijin Road 94, 300071 Tianjin, China; 2 Tianjin State Key Laboratory of Modern Chinese Medicine, Tianjin University of Traditional Chinese Medicine, Poyang Lake Road 10, 301617 Tianjin, China; 3 College of Life and Geographic Sciences, Qinghai Normal University, 36 Wusixi Street, 810008 Qinghai, China; 4 School of Chinese Materia Medica, Tianjin University of Traditional Chinese Medicine, Poyang Lake Road 10, 301617 Tianjin, China

Correction to: *Scientific Reports* 10.1038/s41598-019-47008-8, published online 22 July 2019

The Acknowledgements section in this Article is incomplete.

“This work was supported by grants from the Natural Science Foundation of Tianjin (No. 18JCQNJC14000), the Tianjin City High School Science & Technology Fund Planning Project (No. 20130203), Qinghai Science and Technology Project (No. 2014-HZ-815) and the Ph.D. Candidate Research Innovation Fund of Nankai University. We thank the Guangzhou Gene Denovo Biotechnology Company for assisting with the sequencing analysis.”

should read:

“This work was supported by grants from the Natural Science Foundation of Tianjin (No. 18JCQNJC14000), the Tianjin City High School Science & Technology Fund Planning Project (No. 20130203), the National Natural Science Foundation of China (No. 81303303), Qinghai Science and Technology Project (No. 2014-HZ-815) and the Ph.D. Candidate Research Innovation Fund of Nankai University. We thank the Guangzhou Gene Denovo Biotechnology Company for assisting with the sequencing analysis.”

