# Supplementary material for: Comparison of Four Complete Chloroplast Genomes of Medicinal and Ornamental Meconopsis Species: Genome Organization and Species Discrimination
Source: Sci Rep. 2019 Jul 22;9:10567. doi: 10.1038/s41598-019-47008-8 (PMC6646306; doi:10.1038/s41598-019-47008-8)

Supplementary

Title: Comparison of Four Complete Chloroplast Genomes of Medicinal and Ornamental *Meconopsis* Species: Genome Organization and Species Discrimination

Xiaoxue Li [1+], Wei Tan[2+], Jiqi Sun[1], Junhua Du[3], Chenguang Zheng[1], Xiaoxuan Tian[2], Min Zheng[1], Beibei Xiang [4,*], and Yong Wang[1,*]

[1] College of Life Science, Nankai University, Weijin road 94, 300071 Tianjin, China

[2] Tianjin State Key Laboratory of Modern Chinese Medicine, Tianjin University of Traditional Chinese Medicine, Poyang lake road 10, 301617, Tianjin, China

[3] College of Life and Geographic Sciences, Qinghai Normal University, 36 wusixi Street, 810008, Qinghai, China

[4]School of Chinese Materia Medica, Tianjin University of Traditional Chinese Medicine, Poyang lake road 10, 301617, Tianjin, China

[*]Corresponding: xiangbeibei03230@163.com(B.X.);
wangyong@nankai.edu.cn(Y.W.)

[+]These authors contributed equally to this work

Supplementary Table 1. Primers of the *Meconopsis integrifolia* (Maxim.) Franch to validated the gaps.

Species	Forward sequence	Reverse sequence	Length (bp)
Meconopsis integrifolia (Maxim.) Franch	TCCATGAGATTCATAGTTGCATTAC	GTTTTTCAGGAATAGTGGCGTT	390

Supplementary Table 2. The condon-anticodon recognition pattern and codon usage for the *Meconopsis racemosa* chloroplast genome.

Codon	Amino Acid	No.	RSCU	tRNA	Codon	Amino Acid	No.	RSCU
UUU	F	1004	1.22		UUU	F	7	0.64
UUC	F	643	0.78	trnA-UGC	UUC	F	15	1.36
UUA	L	681	1.63		UUA	L	4	0.37
UUG	L	523	1.25	trnA-UGC	UUG	L	15	1.38
CUU	L	500	1.19		CUU	L	16	1.48
CUC	L	245	0.59	trnC-GCA	CUC	L	12	1.11
CUA	L	350	0.84	trnD-GUC	CUA	L	8	0.74
CUG	L	212	0.51		CUG	L	10	0.92
AUU	I	933	1.36	trnE-UUC	AUU	I	10	1.03
AUC	I	527	0.77		AUC	I	12	1.24
AUA	I	597	0.87	trnF-GAA	AUA	I	7	0.72
AUG	M	547	1	trnfM-CAU	AUG	M	9	1
GUU	V	489	1.39		GUU	V	17	1.17
GUC	V	230	0.65	trnG-GCC	GUC	V	11	0.76
GUA	V	472	1.34		GUA	V	16	1.1
GUG	V	216	0.61	trnG-UCC	GUG	V	14	0.97

CCU	P	329	1.28	trnH-GUG	CCU	P	16	1.36
CCC	P	223	0.87		CCC	P	12	1.02
CCA	P	308	1.2	trnI-CAU	CCA	P	8	0.68
CCG	P	166	0.65		CCG	P	11	0.94
ACU	T	405	1.33	trnI-CAU	ACU	T	4	0.73
ACC	T	269	0.88	trnI-GAU	ACC	T	8	1.45
ACA	T	382	1.25		ACA	T	4	0.73
ACG	T	166	0.54	trnI-GAU	ACG	T	6	1.09
GCU	A	451	1.64		GCU	A	13	1.27
GCC	A	196	0.71	trnK-UUU	GCC	A	8	0.78
GCA	A	312	1.14	trnL-CAA	GCA	A	9	0.88
GCG	A	140	0.51		GCG	A	11	1.07
UAU	Y	795	1.44	trnL-CAA	UAU	Y	4	1.14
UAC	Y	306	0.56		UAC	Y	3	0.86
CAU	H	466	1.38	trnL-UAA	CAU	H	13	1.73
CAC	H	207	0.62	trnL-UAG	CAC	H	2	0.27
CAA	Q	659	1.44		CAA	Q	10	0.91
CAG	Q	258	0.56	trnM-CAU	CAG	Q	12	1.09
AAU	N	810	1.41	trnN-GUU	AAU	N	12	1.14
AAC	N	339	0.59		AAC	N	9	0.86
AAA	K	1009	1.4	trnN-GUU	AAA	K	10	1.33
AAG	K	428	0.6		AAG	K	5	0.67
GAU	D	664	1.49	trnP-UGG	GAU	D	13	1.37
GAC	D	227	0.51	trnQ-UUG	GAC	D	6	0.63
GAA	E	844	1.42		GAA	E	13	0.79

GAG	E	343	0.58	trnR-ACG	GAG	E	20	1.21
UGU	C	282	1.23		UGU	C	12	1.2
UGC	C	175	0.77	trnR-ACG	UGC	C	8	0.8
UGG	W	484	1		UGG	W	13	1
CGU	R	249	0.89	trnR-UCU	CGU	R	9	0.82
CGC	R	107	0.38	trnS-GCU	CGC	R	8	0.73
CGA	R	335	1.19		CGA	R	12	1.09
CGG	R	149	0.53	trnS-GGA	CGG	R	14	1.27
AGA	R	571	2.03		AGA	R	9	0.82
AGG	R	277	0.98	trnS-UGA	AGG	R	14	1.27
GGU	G	468	1.15	trnT-GGU	GGU	G	27	1.52
GGC	G	201	0.5		GGC	G	15	0.85
GGA	G	583	1.44	trnT-UGU	GGA	G	14	0.79
GGG	G	371	0.91		GGG	G	15	0.85
UCU	S	574	1.57	trnV-GAC	UCU	S	9	0.61
UCC	S	340	0.93	trnV-GAC	UCC	S	12	0.82
UCA	S	477	1.31		UCA	S	17	1.16
UCG	S	240	0.66	trnV-UAC	UCG	S	18	1.23
AGU	S	355	0.97	trnW-CCA	AGU	S	13	0.89
AGC	S	203	0.56		AGC	S	19	1.3
UAA	STOP	367	1.07	trnY-GUA	UAA	STOP	12	1.29
UAG	STOP	265	0.77		UAG	STOP	12	1.29
UGA	STOP	394	1.15		UGA	STOP	4	0.43

Supplementary Table 3. The condon-anticodon recognition pattern and codon usage for the *Meconopsis integrifolia(Maxim.) Franch* chloroplast

genome.

Codon	Amino Acid	No.	RSCU	tRNA	Codon	Amino Acid	No.	RSCU
UUU	F	938	1.25		UUU	F	7	0.61
UUC	F	565	0.75	trnA-UGC	UUC	F	16	1.39
UUA	L	822	1.69		UUA	L	3	0.29
UUG	L	587	1.21	trnA-UGC	UUG	L	13	1.26
CUU	L	586	1.2	trnC-GCA	CUU	L	14	1.35
CUC	L	256	0.53		CUC	L	16	1.55
CUA	L	407	0.84	trnD-GUC	CUA	L	7	0.68
CUG	L	260	0.53	trnE-UUC	CUG	L	9	0.87
AUU	I	979	1.36		AUU	I	13	1.22
AUC	I	449	0.62	trnF-GAA	AUC	I	11	1.03
AUA	I	738	1.02		AUA	I	8	0.75
AUG	M	646	1	trnfM-CAU	AUG	M	7	1
GUU	V	523	1.38	trnG-GCC	GUU	V	14	1
GUC	V	216	0.57		GUC	V	11	0.79
GUA	V	538	1.42	trnG-UCC	GUA	V	17	1.21
GUG	V	240	0.63		GUG	V	14	1
CCU	P	394	1.42	trnH-GUG	CCU	P	16	1.39
CCC	P	207	0.75	trnI-CAU	CCC	P	11	0.96
CCA	P	333	1.2		CCA	P	9	0.78
CCG	P	175	0.63	trnI-CAU	CCG	P	10	0.87
ACU	T	449	1.4		ACU	T	5	1.05
ACC	T	239	0.75	trnI-GAU	ACC	T	7	1.47

ACA	T	408	1.28	trnI-GAU	ACA	T	4	0.84
ACG	T	183	0.57		ACG	T	3	0.63
GCU	A	525	1.65	trnK-UUU	GCU	A	14	1.27
GCC	A	224	0.7		GCC	A	9	0.82
GCA	A	371	1.16	trnL-CAA	GCA	A	9	0.82
GCG	A	155	0.49	trnL-CAA	GCG	A	12	1.09
UAU	Y	769	1.58		UAU	Y	5	1.11
UAC	Y	207	0.42	trnL-UAA	UAC	Y	4	0.89
CAU	H	482	1.5		CAU	H	12	1.6
CAC	H	160	0.5	trnL-UAG	CAC	H	3	0.4
CAA	Q	664	1.46	trnM-CAU	CAA	Q	11	0.96
CAG	Q	245	0.54		CAG	Q	12	1.04
AAU	N	886	1.46	trnN-GUU	AAU	N	13	1.3
AAC	N	331	0.54		AAC	N	7	0.7
AAA	K	1058	1.41	trnN-GUU	AAA	K	10	1.33
AAG	K	447	0.59	trnP-UGG	AAG	K	5	0.67
GAU	D	787	1.54		GAU	D	11	1.47
GAC	D	238	0.46	trnQ-UUG	GAC	D	4	0.53
GAA	E	928	1.41	trnR-ACG	GAA	E	13	0.81
GAG	E	387	0.59		GAG	E	19	1.19
UGU	C	222	1.34	trnR-ACG	UGU	C	12	1.14
UGC	C	110	0.66		UGC	C	9	0.86
UGG	W	493	1	trnR-UCU	UGG	W	17	1
CGU	R	299	1.16	trnS-GCU	CGU	R	8	0.71
CGC	R	100	0.39		CGC	R	7	0.62

CGA	R	317	1.23	trnS-GGA	CGA	R	9	0.79
CGG	R	142	0.55		CGG	R	15	1.32
AGA	R	490	1.9	trnS-UGA	AGA	R	12	1.06
AGG	R	202	0.78	trnT-GGU	AGG	R	17	1.5
GGU	G	506	1.22		GGU	G	28	1.49
GGC	G	167	0.4	trnT-UGU	GGC	G	15	0.8
GGA	G	652	1.57		GGA	G	15	0.8
GGG	G	338	0.81	trnV-GAC	GGG	G	17	0.91
UCU	S	519	1.53	trnV-GAC	UCU	S	9	0.64
UCC	S	371	1.1		UCC	S	14	1
UCA	S	442	1.31	trnV-UAC	UCA	S	16	1.14
UCG	S	208	0.61		UCG	S	16	1.14
AGU	S	348	1.03	trnW-CCA	AGU	S	10	0.71
AGC	S	144	0.43	trnY-GUA	AGC	S	19	1.36
UAA	STOP	103	1.05		UAA	STOP	11	1.18
UAG	STOP	103	1.05		UAG	STOP	11	1.18
UGA	STOP	87	0.89		UGA	STOP	6	0.64

Supplementary Table 4. The condon-anticodon recognition pattern and codon usage for the *Meconopsis punice* chloroplast genome.

Codon	Amino Acid	No.	RSCU	tRNA	Codon	Amino Acid	No.	RSCU
UUU	F	944	1.28	trnA-UGC	UUU	F	8	0.59
UUC	F	532	0.72		UUC	F	19	1.41

UUA	L	828	1.82	trnA-UGC	UUA	L	10	0.9
UUG	L	539	1.18		UUG	L	16	1.43
CUU	L	592	1.3	trnC-GCA	CUU	L	3	0.27
CUC	L	206	0.45		CUC	L	18	1.61
CUA	L	373	0.82	trnD-GUC	CUA	L	8	0.72
CUG	L	196	0.43	trnE-UUC	CUG	L	12	1.07
AUU	I	1048	1.43		AUU	I	11	1.06
AUC	I	428	0.58	trnF-GAA	AUC	I	13	1.26
AUA	I	724	0.99		AUA	I	7	0.68
AUG	M	632	1	trnfM-CAU	AUG	M	9	1
GUU	V	528	1.45	trnG-GCC	GUU	V	18	1.31
GUC	V	201	0.55		GUC	V	15	1.09
GUA	V	514	1.42	trnG-UCC	GUA	V	12	0.87
GUG	V	210	0.58		GUG	V	10	0.73
CCU	P	427	1.53	trnH-GUG	CCU	P	12	1.12
CCC	P	216	0.77	trnI-CAU	CCC	P	12	1.12
CCA	P	321	1.15		CCA	P	6	0.56
CCG	P	155	0.55	trnI-CAU	CCG	P	13	1.21
ACU	T	504	1.5	trnI-GAU	ACU	T	8	1.33
ACC	T	258	0.77		ACC	T	5	0.83
ACA	T	418	1.24	trnI-GAU	ACA	T	3	0.5
ACG	T	166	0.49		ACG	T	8	1.33
GCU	A	595	1.74	trnK-UUU	GCU	A	11	0.92
GCC	A	238	0.7	trnL-CAA	GCC	A	11	0.92
GCA	A	385	1.13		GCA	A	12	1

GCG	A	150	0.44	trnL-CAA	GCG	A	14	1.17
UAU	Y	784	1.61		UAU	Y	3	0.75
UAC	Y	187	0.39	trnL-UAA	UAC	Y	5	1.25
CAU	H	490	1.52		CAU	H	8	1.6
CAC	H	154	0.48	trnL-UAG	CAC	H	2	0.4
CAA	Q	691	1.52	trnM-CAU	CAA	Q	13	1.04
CAG	Q	221	0.48		CAG	Q	12	0.96
AAU	N	942	1.52	trnN-GUU	AAU	N	15	1.58
AAC	N	298	0.48		AAC	N	4	0.42
AAA	K	1079	1.47	trnN-GUU	AAA	K	12	1
AAG	K	390	0.53	trnP-UGG	AAG	K	12	1
GAU	D	847	1.54		GAU	D	8	1.23
GAC	D	253	0.46	trnQ-UUG	GAC	D	5	0.77
GAA	E	998	1.45		GAA	E	9	0.72
GAG	E	377	0.55	trnR-ACG	GAG	E	16	1.28
UGU	C	221	1.42	trnR-ACG	UGU	C	8	1.23
UGC	C	90	0.58		UGC	C	5	0.77
UGG	W	467	1	trnR-UCU	UGG	W	29	1
CGU	R	343	1.3	trnS-GCU	CGU	R	8	0.66
CGC	R	100	0.38		CGC	R	11	0.9
CGA	R	347	1.32	trnS-GGA	CGA	R	11	0.9
CGG	R	120	0.46		CGG	R	17	1.4
AGA	R	487	1.85	trnS-UGA	AGA	R	9	0.74
AGG	R	182	0.69	trnT-GGU	AGG	R	17	1.4
GGU	G	570	1.28		GGU	G	20	1.45

GGC	G	181	0.4	trnT-UGU	GGC	G	12	0.87
GGA	G	704	1.57		GGA	G	9	0.65
GGG	G	333	0.74	trnV-GAC	GGG	G	14	1.02
UCU	S	558	1.62		UCU	S	9	0.75
UCC	S	354	1.02	trnV-GAC	UCC	S	15	1.25
UCA	S	444	1.29	trnV-UAC	UCA	S	15	1.25
UCG	S	184	0.53		UCG	S	9	0.75
AGU	S	404	1.17	trnW-CCA	AGU	S	12	1
AGC	S	129	0.37		AGC	S	12	1
UAA	STOP	42	1.48	trnY-GUA	UAA	STOP	7	0.57
UAG	STOP	23	0.81		UAG	STOP	20	1.62
UGA	STOP	20	0.71		UGA	STOP	10	0.81

Supplementary Table 5. The condon-anticodon recognition pattern and codon usage for the *Meconopsis horridula* chloroplast genome.

Codon	Amino Acid	No.	RSCU	tRNA	Codon	Amino Acid	No.	RSCU
UUU	F	925	1.21		UUU	F	7	0.61
UUC	F	600	0.79	trnA-UGC	UUC	F	16	1.39
UUA	L	775	1.64	trnA-UGC	UUA	L	3	0.29
UUG	L	603	1.27		UUG	L	13	1.26
CUU	L	574	1.21	trnC-GCA	CUU	L	14	1.35
CUC	L	246	0.52		CUC	L	16	1.55
CUA	L	408	0.86	trnD-GUC	CUA	L	7	0.68
CUG	L	236	0.5	trnE-UUC	CUG	L	9	0.87
AUU	I	975	1.31		AUU	I	13	1.22

AUC	I	542	0.73	trnF-GAA	AUC	I	11	1.03
AUA	I	721	0.97		AUA	I	8	0.75
AUG	M	650	1	trnfM-CAU	AUG	M	7	1
GUU	V	509	1.35		GUU	V	14	1
GUC	V	222	0.59	trnG-GCC	GUC	V	11	0.79
GUA	V	537	1.43	trnG-UCC	GUA	V	17	1.21
GUG	V	239	0.63		GUG	V	14	1
CCU	P	370	1.34	trnH-GUG	CCU	P	16	1.39
CCC	P	225	0.82		CCC	P	11	0.96
CCA	P	334	1.21	trnI-CAU	CCA	P	9	0.78
CCG	P	172	0.62	trnI-CAU	CCG	P	10	0.87
ACU	T	449	1.43		ACU	T	5	1.05
ACC	T	248	0.79	trnI-GAU	ACC	T	7	1.47
ACA	T	398	1.27		ACA	T	4	0.84
ACG	T	157	0.5	trnI-GAU	ACG	T	3	0.63
GCU	A	501	1.65	trnK-UUU	GCU	A	14	1.27
GCC	A	211	0.69		GCC	A	9	0.82
GCA	A	356	1.17	trnL-CAA	GCA	A	9	0.82
GCG	A	147	0.48	trnL-CAA	GCG	A	12	1.09
UAU	Y	749	1.53		UAU	Y	4	1
UAC	Y	230	0.47	trnL-UAA	UAC	Y	4	1
CAU	H	464	1.44		CAU	H	13	1.63
CAC	H	181	0.56	trnL-UAG	CAC	H	3	0.38
CAA	Q	690	1.42	trnM-CAU	CAA	Q	11	0.96
CAG	Q	283	0.58		CAG	Q	12	1.04

AAU	N	847	1.46	trnN-GUU	AAU	N	13	1.3
AAC	N	310	0.54		AAC	N	7	0.7
AAA	K	1028	1.4	trnN-GUU	AAA	K	10	1.33
AAG	K	439	0.6		AAG	K	5	0.67
GAU	D	707	1.5	trnP-UGG	GAU	D	11	1.47
GAC	D	233	0.5	trnQ-UUG	GAC	D	4	0.53
GAA	E	881	1.41		GAA	E	13	0.81
GAG	E	369	0.59	trnR-ACG	GAG	E	19	1.19
UGU	C	237	1.32		UGU	C	12	1.14
UGC	C	121	0.68	trnR-ACG	UGC	C	9	0.86
UGG	W	458	1	trnR-UCU	UGG	W	16	1
CGU	R	292	1.11		CGU	R	8	0.7
CGC	R	103	0.39	trnS-GCU	CGC	R	7	0.61
CGA	R	339	1.29		CGA	R	9	0.78
CGG	R	154	0.59	trnS-GGA	CGG	R	16	1.39
AGA	R	474	1.8	trnS-UGA	AGA	R	12	1.04
AGG	R	217	0.82		AGG	R	17	1.48
GGU	G	523	1.25	trnT-GGU	GGU	G	28	1.47
GGC	G	181	0.43	trnT-UGU	GGC	G	16	0.84
GGA	G	627	1.5		GGA	G	15	0.79
GGG	G	340	0.81	trnV-GAC	GGG	G	17	0.89
UCU	S	507	1.6		UCU	S	9	0.65
UCC	S	321	1.01	trnV-GAC	UCC	S	14	1.01
UCA	S	403	1.27	trnV-UAC	UCA	S	16	1.16
UCG	S	200	0.63		UCG	S	16	1.16

AGU	S	343	1.08	trnW-CCA	AGU	S	11	0.8
AGC	S	133	0.42		AGC	S	17	1.23
UAA	STOP	217	1.04	trnY-GUA	UAA	STOP	11	1.18
UAG	STOP	209	1.01		UAG	STOP	11	1.18
UGA	STOP	197	0.95		UGA	STOP	6	0.64

Supplementary Table 6. RNA editing sites predicted in the chloroplast genome of *Meconopsis racemosa*.

**Gene Name**	**Length**	**Strand**	**Region**	**Nt pos**	**AA pos**	**Align Col**	**Effect**
accD	1461	+	LSC	794	265	288	TCG (S) => TTG (L)
accD	1461	+	LSC	1157	386	421	TCA (S) => TTA (L)
atpA	1524	−	LSC	773	258	258	TCA (S) => TTA (L)
atpA	1524	−	LSC	791	264	264	CCC (P) => CTC (L)
atpB	1497	−	LSC	1184	395	395	TCA (S) => TTA (L)
atpF	555	−	LSC	92	31	31	CCA (P) => CTA (L)
atpI	744	−	LSC	349	117	120	CGG (R) => TGG (W)

atpI	744	−	LSC	629	210	213	TCA (S) => TTA (L)
ccsA	975	+	SSC	634	212	221	CAC (H) => TAC (Y)
clpP	609	−	LSC	224	75	75	ACA (T) => ATA (I)
clpP	609	−	LSC	559	187	187	CAT (H) => TAT (Y)
matK	1527	−	LSC	307	103	110	CTT (L) => TTT (F)
matK	1527	−	LSC	445	149	159	CAT (H) => TAT (Y)
matK	1527	−	LSC	469	157	167	CAC (H) => TAC (Y)
matK	1527	−	LSC	655	219	229	CAT (H) => TAT (Y)
matK	1527	−	LSC	1000	334	344	CTT (L) => TTT (F)
matK	1527	−	LSC	1426	476	486	CCT (P) => TCT (S)
ndhA	1089	−	SSC	107	36	36	CCT (P) => CTT (L)
ndhA	1089	−	SSC	341	114	114	TCA (S) => TTA (L)
ndhA	1089	−	SSC	566	189	189	TCA (S) => TTA (L)

ndhB	1533	+	IR	149	50	50	TCA (S) => TTA (L)
ndhB	1533	+	IR	467	156	156	CCA (P) => CTA (L)
ndhB	1533	+	IR	542	181	181	ACG (T) => ATG (M)
ndhB	1533	+	IR	586	196	196	CAT (H) => TAT (Y)
ndhB	1533	+	IR	611	204	204	TCA (S) => TTA (L)
ndhB	1533	+	IR	737	246	246	CCA (P) => CTA (L)
ndhB	1533	+	IR	746	249	249	TCT (S) => TTT (F)
ndhB	1533	+	IR	830	277	277	TCA (S) => TTA (L)
ndhB	1533	+	IR	836	279	279	TCA (S) => TTA (L)
ndhB	1533	+	IR	902	301	301	ACC (T) => ATC (I)
ndhB	1533	+	IR	1255	419	419	CAT (H) => TAT (Y)
ndhB	1533	+	IR	1481	494	494	CCA (P) => CTA (L)
ndhD	1503	−	SSC	985	329	329	CCC (P) => TCC (S)

ndhD	1503	–	SSC	1081	361	361	CCA (P) => TCA (S)
ndhD	1503	–	SSC	1150	384	384	CAT (H) => TAT (Y)
ndhD	1503	–	SSC	1414	472	479	CTT (L) => TTT (F)
ndhF	2219	–	SSC	128	43	43	CCA (P) => CTA (L)
ndhF	2219	–	SSC	185	62	63	CCT (P) => CTT (L)
ndhF	2219	–	SSC	193	65	66	CCG (P) => TCG (S)
ndhF	2219	–	SSC	305	102	106	ACA (T) => ATA (I)
ndhF	2219	–	SSC	383	128	132	ACG (T) => ATG (M)
ndhF	2219	–	SSC	511	171	175	CCT (P) => TCT (S)
ndhF	2219	–	SSC	521	174	178	TCA (S) => TTA (L)
ndhF	2219	–	SSC	902	301	305	CCT (P) => CTT (L)
ndhF	2219	–	SSC	953	318	326	TCG (S) => TTG (L)
ndhF	2219	–	SSC	965	322	330	TCA (S) => TTA (L)

ndhF	2219	−	SSC	1106	369	377	CCA (P) => CTA (L)
ndhF	2219	−	SSC	1138	380	388	CCG (P) => TCG (S)
ndhF	2219	−	SSC	1282	428	438	CTT (L) => TTT (F)
ndhF	2219	−	SSC	1402	468	478	CCC (P) => TTC (F)
ndhF	2219	−	SSC	1403	468	478	CCC (P) => TTC (F)
ndhF	2219	−	SSC	1861	621	641	CCT (P) => TCT (S)
ndhG	531	−	SSC	148	50	50	CAT (H) => TAT (Y)
ndhG	531	−	SSC	155	52	52	CCA (P) => CTA (L)
ndhG	531	−	SSC	166	56	56	CAT (H) => TAT (Y)
ndhG	531	−	SSC	314	105	105	ACA (T) => ATA (I)
petB	648	+	LSC	418	140	140	CGG (R) => TGG (W)
petB	648	+	LSC	611	204	204	CCA (P) => CTA (L)
psbE	252	−	LSC	200	67	67	TCG (S) => TTG (L)

psbF	120	−	LSC	77	26	26	TCT (S) => TTT (F)
rpl2	825	+	IR	19	7	12	CCA (P) => TCA (S)
rpl2	825	+	IR	524	175	182	CCC (P) => CTC (L)
rpl2	825	+	IR	575	192	199	GCT (A) => GTT (V)
rpl20	354	−	LSC	256	86	86	CAC (H) => TAC (Y)
rpl20	354	−	LSC	308	103	103	TCA (S) => TTA (L)
rpoA	1035	−	LSC	368	123	123	TCA (S) => TTA (L)
rpoA	1035	−	LSC	830	277	279	TCA (S) => TTA (L)
rpoB	3213	−	LSC	473	158	159	TCA (S) => TTA (L)
rpoB	3213	−	LSC	566	189	190	TCG (S) => TTG (L)
rpoB	3213	−	LSC	623	208	209	CCG (P) => CTG (L)
rpoB	3213	−	LSC	2426	809	827	TCA (S) => TTA (L)
rpoB	3213	−	LSC	2498	833	851	ACT (T) => ATT (I)

rpoC1	2049	−	LSC	41	14	14	TCA (S) => TTA (L)
rpoC2	4164	−	LSC	1493	498	517	ACG (T) => ATG (M)
rpoC2	4164	−	LSC	1586	529	548	TCT (S) => TTT (F)
rpoC2	4164	−	LSC	1774	592	613	CGT (R) => TGT (C)
rpoC2	4164	−	LSC	2293	765	950	CGG (R) => TGG (W)
rpoC2	4164	−	LSC	2792	931	1124	TCG (S) => TTG (L)
rpoC2	4164	−	LSC	2818	940	1133	CCT (P) => TCT (S)
rpoC2	4164	−	LSC	3728	1243	1455	TCA (S) => TTA (L)
rps2	711	+	LSC	248	83	83	TCA (S) => TTA (L)
rps2	711	+	LSC	314	105	105	TCA (S) => TTA (L)
rps8	399	−	LSC	43	15	15	CCG (P) => TCG (S)
rps8	399	−	LSC	122	41	42	TCG (S) => TTG (L)
rps8	399	−	LSC	326	109	113	TCG (S) => TTG (L)

rps8	399	−	LSC	392	131	135	CCT (P) => CTT (L)
rps8	399	−	LSC	397	133	137	CAT (H) => TAT (Y)
rps14	303	−	LSC	149	50	53	CCA (P) => CTA (L)
rps16	267	−	LSC	143	48	48	TCA (S) => TTA (L)
rps16	267	−	LSC	212	71	71	TCA (S) => TTA (L)
ycf3	507	−	LSC	44	15	15	TCT (S) => TTT (F)
ycf3	507	−	LSC	407	136	136	TCC (S) => TTC (F)

Supplementary Table 7. RNA editing sites predicted in the chloroplast genome of *Meconopsis integrifolia* (Maxim.) Franch.

**Gene Name**	**Length**	**Strand**	**Region**	**Nt pos**	**AA pos**	**Align Col**	**Effect**
accD	1479	+	LSC	812	271	293	TCG (S) => TTG (L)
accD	1479	+	LSC	1175	392	426	TCA (S) => TTA (L)
atpA	1524	−	LSC	773	258	258	TCA (S) => TTA (L)

atpB	1497	−	LSC	1184	395	395	TCA (S) => TTA (L)
atpF	555	−	LSC	92	31	31	CCA (P) => CTA (L)
ccsA	969	+	SSC	634	212	221	CAC (H) => TAC (Y)
clpP	609	−	LSC	224	75	75	ACA (T) => ATA (I)
clpP	609	−	LSC	559	187	187	CAT (H) => TAT (Y)
matK	1527	−	LSC	307	103	110	CTT (L) => TTT (F)
matK	1527	−	LSC	445	149	159	CAT (H) => TAT (Y)
matK	1527	−	LSC	469	157	167	CAC (H) => TAC (Y)
matK	1527	−	LSC	655	219	229	CAT (H) => TAT (Y)
matK	1527	−	LSC	1000	334	344	CTT (L) => TTT (F)
matK	1527	−	LSC	1426	476	486	CCT (P) => TCT (S)
ndhA	1089	−	SSC	107	36	36	CCT (P) => CTT (L)

ndhA	1089	−	SSC	341	114	114	TCA (S) => TTA (L)
ndhA	1089	−	SSC	566	189	189	TCA (S) => TTA (L)
ndhB	1533	+	IR	149	50	50	TCA (S) => TTA (L)
ndhB	1533	+	IR	467	156	156	CCA (P) => CTA (L)
ndhB	1533	+	IR	542	181	181	ACG (T) => ATG (M)
ndhB	1533	+	IR	586	196	196	CAT (H) => TAT (Y)
ndhB	1533	+	IR	611	204	204	TCA (S) => TTA (L)
ndhB	1533	+	IR	737	246	246	CCA (P) => CTA (L)
ndhB	1533	+	IR	746	249	249	TCT (S) => TTT (F)
ndhB	1533	+	IR	830	277	277	TCA (S) => TTA (L)
ndhB	1533	+	IR	836	279	279	TCA (S) => TTA (L)
ndhB	1533	+	IR	902	301	301	ACC (T) => ATC (I)

ndhB	1533	+	IR	1255	419	419	CAT (H) => TAT (Y)
ndhB	1533	+	IR	1481	494	494	CCA (P) => CTA (L)
ndhD	1503	−	SSC	2	1	1	ACG (T) => ATG (M)
ndhD	1503	−	SSC	313	105	105	CGG (R) => TGG (W)
ndhD	1503	−	SSC	383	128	128	CCT (P) => CTT (L)
ndhD	1503	−	SSC	887	296	296	CCT (P) => CTT (L)
ndhD	1503	−	SSC	947	316	316	ACA (T) => ATA (I)
ndhD	1503	−	SSC	1298	433	433	TCA (S) => TTA (L)
ndhD	1503	−	SSC	1310	437	437	TCA (S) => TTA (L)
ndhF	2220	−	SSC	392	131	131	TCC (S) => TTC (F)
ndhF	2220	−	SSC	586	196	196	CTT (L) => TTT (F)
ndhF	2220	−	SSC	623	208	208	TCG (S) => TTG (L)

ndhF	2220	−	SSC	1385	462	462	TCT (S) => TTT (F)
ndhF	2220	−	SSC	1567	523	526	CTT (L) => TTT (F)
ndhF	2220	−	SSC	1846	616	625	CTT (L) => TTT (F)
ndhF	2220	−	SSC	2177	726	735	TCA (S) => TTA (L)
ndhG	531	−	SSC	148	50	50	CAT (H) => TAT (Y)
ndhG	531	−	SSC	155	52	52	CCA (P) => CTA (L)
ndhG	531	−	SSC	166	56	56	CAT (H) => TAT (Y)
ndhG	531	−	SSC	314	105	105	ACA (T) => ATA (I)
petB	648	+	LSC	418	140	140	CGG (R) => TGG (W)
petB	648	+	LSC	611	204	204	CCA (P) => CTA (L)
psbE	252	−	LSC	214	72	72	CCT (P) => TCT (S)
psbF	120	−	LSC	77	26	26	TCT (S) => TTT (F)

rpl2	825	+	IR	2	1	1	ACG (T) => ATG (M)
rpl20	354	−	LSC	256	86	86	CAC (H) => TAC (Y)
rpl20	354	−	LSC	308	103	103	TCA (S) => TTA (L)
rpoA	1035	−	LSC	368	123	123	TCA (S) => TTA (L)
rpoA	1035	−	LSC	830	277	279	TCA (S) => TTA (L)
rpoB	3213	−	LSC	473	158	159	TCA (S) => TTA (L)
rpoB	3213	−	LSC	566	189	190	TCG (S) => TTG (L)
rpoB	3213	−	LSC	623	208	209	CCG (P) => CTG (L)
rpoB	3213	−	LSC	2248	750	768	CCA (P) => TCA (S)
rpoB	3213	−	LSC	2426	809	827	TCA (S) => TTA (L)
rpoC1	2049	−	LSC	41	14	14	TCA (S) => TTA (L)
rpoC1	2049	−	LSC	959	320	337	TCA (S) => TTA (L)

rpoC1	2049	–	LSC	1447	483	500	CGC (R) => TGC (C)
rpoC2	4158	–	LSC	1493	498	517	ACG (T) => ATG (M)
rpoC2	4158	–	LSC	1586	529	548	TCT (S) => TTT (F)
rpoC2	4158	–	LSC	1774	592	613	CGT (R) => TGT (C)
rpoC2	4158	–	LSC	2287	763	948	CGG (R) => TGG (W)
rpoC2	4158	–	LSC	2812	938	1131	CCT (P) => TCT (S)
rpoC2	4158	–	LSC	3722	1241	1453	TCA (S) => TTA (L)
rps2	711	–	LSC	248	83	83	TCA (S) => TTA (L)
rps2	711	–	LSC	314	105	105	TCA (S) => TTA (L)
rps8	399	–	LSC	182	61	61	TCA (S) => TTA (L)
rps14	303	–	LSC	149	50	53	CCA (P) => CTA (L)
rps16	267	–	LSC	143	48	48	TCA (S) => TTA (L)

rps16	267	−	LSC	212	71	71	TCA (S) => TTA (L)
ycf3	507	−	LSC	44	15	15	TCT (S) => TTT (F)
ycf3	507	−	LSC	407	136	136	TCC (S) => TTC (F)

Supplementary Table 8. RNA editing sites predicted in the chloroplast genome of *Meconopsis horridula*.

**Gene Name**	**Length**	**Strand**	**Region**	**Nt pos**	**AA pos**	**Align Col**	**Effect**
accD	1461	+	LSC	794	265	288	TCG (S) => TTG (L)
accD	1461	+	LSC	1157	386	421	TCA (S) => TTA (L)
atpA	1524	−	LSC	773	258	258	TCA (S) => TTA (L)
atpA	1524	−	LSC	791	264	264	CCC (P) => CTC (L)
atpB	1497	−	LSC	1184	395	395	TCA (S) => TTA (L)
atpF	555	−	LSC	92	31	31	CCA (P) => CTA (L)
atpI	744	−	LSC	349	117	120	CGG (R) => TGG (W)

atpI	744	−	LSC	629	210	213	TCA (S) => TTA (L)
ccsA	975	+	SSC	634	212	221	CAC (H) => TAC (Y)
clpP	609	−	LSC	224	75	75	ACA (T) => ATA (I)
clpP	609	−	LSC	559	187	187	CAT (H) => TAT (Y)
matK	1527	−	LSC	52	18	18	CTT (L) => TTT (F)
matK	1527	−	LSC	283	95	95	CTT (L) => TTT (F)
matK	1527	−	LSC	362	121	124	CCC (P) => CTC (L)
matK	1527	−	LSC	479	160	163	ACT (T) => ATT (I)
matK	1527	−	LSC	650	217	220	TCC (S) => TTC (F)
matK	1527	−	LSC	715	239	242	CCA (P) => TCA (S)
ndhA	1089	−	SSC	107	36	36	CCT (P) => CTT (L)
ndhA	1089	−	SSC	341	114	114	TCA (S) => TTA (L)
ndhA	1089	−	SSC	566	189	189	TCA (S) => TTA (L)
ndhB	1533	−	IR	149	50	50	TCA (S) => TTA (L)

ndhB	1533	–	IR	467	156	156	CCA (P) => CTA (L)
ndhB	1533	–	IR	542	181	181	ACG (T) => ATG (M)
ndhB	1533	–	IR	586	196	196	CAT (H) => TAT (Y)
ndhB	1533	–	IR	611	204	204	TCA (S) => TTA (L)
ndhB	1533	–	IR	737	246	246	CCA (P) => CTA (L)
ndhB	1533	–	IR	746	249	249	TCT (S) => TTT (F)
ndhB	1533	–	IR	830	277	277	TCA (S) => TTA (L)
ndhB	1533	–	IR	836	279	279	TCA (S) => TTA (L)
ndhB	1533	–	IR	902	301	301	ACC (T) => ATC (I)
ndhB	1533	–	IR	1255	419	419	CAT (H) => TAT (Y)
ndhB	1533	–	IR	1481	494	494	CCA (P) => CTA (L)
ndhD	1503	–	SSC	2	1	1	ACG (T) => ATG (M)
ndhD	1503	–	SSC	313	105	105	CGG (R) => TGG (W)

ndhD	1503	–	SSC	383	128	128	CCT (P) => CTT (L)
ndhD	1503	–	SSC	674	225	225	TCA (S) => TTA (L)
ndhD	1503	–	SSC	887	296	296	CCT (P) => CTT (L)
ndhD	1503	–	SSC	947	316	316	ACA (T) => ATA (I)
ndhD	1503	–	SSC	1298	433	433	TCA (S) => TTA (L)
ndhD	1503	–	SSC	1310	437	437	TCA (S) => TTA (L)
ndhF	2219	–	SSC	392	131	131	TCC (S) => TTC (F)
ndhF	2219	–	SSC	586	196	196	CTT (L) => TTT (F)
ndhF	2219	–	SSC	623	208	208	TCG (S) => TTG (L)
ndhF	2219	–	SSC	1385	462	462	TCT (S) => TTT (F)
ndhF	2219	–	SSC	1567	523	526	CTT (L) => TTT (F)
ndhF	2219	–	SSC	1691	564	568	ACT (T) => ATT (I)
ndhF	2219	–	SSC	1846	616	625	CTT (L) => TTT (F)
ndhF	2219	–	SSC	2177	726	735	TCA (S) => TTA (L)
ndhG	531	–	SSC	148	50	50	CAT (H) => TAT (Y)

ndhG	531	−	SSC	155	52	52	CCA (P) => CTA (L)
ndhG	531	−	SSC	166	56	56	CAT (H) => TAT (Y)
ndhG	531	−	SSC	314	105	105	ACA (T) => ATA (I)
petB	648	+	LSC	418	140	140	CGG (R) =>TGG (W)
petB	648	+	LSC	611	204	204	CCA (P) => CTA (L)
psbE	252	−	LSC	214	72	72	CCT (P) => TCT (S)
psbF	120	−	LSC	77	26	26	TCT (S) => TTT (F)
psbL	117	−	LSC	74	25	32	TCT (S) => TTT (F)
rpl2	825	+	IR	2	1	1	ACG (T) => ATG (M)
rpl20	354	−	LSC	256	86	86	CAC (H) => TAC (Y)
rpl20	354	−	LSC	308	103	103	TCA (S) => TTA (L)
rpoA	1035	−	LSC	368	123	123	TCA (S) => TTA (L)
rpoA	1035	−	LSC	830	277	279	TCA (S) => TTA (L)

rpoB	3213	–	LSC	473	158	159	TCA (S) => TTA (L)
rpoB	3213	–	LSC	566	189	190	TCG (S) => TTG (L)
rpoB	3213	–	LSC	623	208	209	CCG (P) => CTG (L)
rpoB	3213	–	LSC	2426	809	827	TCA (S) => TTA (L)
rpoB	3213	–	LSC	2498	833	851	ACT (T) => ATT (I)
rpoC1	2049	–	LSC	41	14	14	TCA (S) => TTA (L)
rpoC1	2049	–	LSC	1946	649	674	ACT (T) => ATT (I)
rpoC2	4164	–	LSC	1493	498	517	ACG (T) => ATG (M)
rpoC2	4164	–	LSC	1586	529	548	TCT (S) => TTT (F)
rpoC2	4164	–	LSC	1774	592	613	CGT (R) => TGT (C)
rpoC2	4164	–	LSC	2293	765	950	CGG (R) => TGG (W)
rpoC2	4164	–	LSC	2792	931	1124	TCG (S) => TTG (L)
rpoC2	4164	–	LSC	2818	940	1133	CCT (P) => TCT (S)
rpoC2	4164	–	LSC	3728	1243	1455	TCA (S) => TTA (L)

rps2	711	−	LSC	248	83	83	TCA (S) => TTA (L)
rps2	711	−	LSC	314	105	105	TCA (S) => TTA (L)
rps8	399	−	LSC	182	61	61	TCA (S) => TTA (L)
rps14	303	−	LSC	149	50	53	CCA (P) => CTA (L)
rps16	267	−	LSC	143	48	48	TCA (S) => TTA (L)
rps16	267	−	LSC	212	71	71	TCA (S) => TTA (L)
ycf3	507	−	LSC	44	15	15	TCT (S) => TTT (F)
ycf3	507	−	LSC	407	136	136	TCC (S) => TTC (F)

Supplementary Table 9. RNA editing sites predicted in the chloroplast genome of *Meconopsis punicea*.

**Gene Name**	**Length**	**Strand**	**Region**	**Nt pos**	**AA pos**	**Align Col**	**Effect**
accD	1461	+	LSC	794	265	288	TCG (S) => TTG (L)
accD	1461	+	LSC	1157	386	421	TCA (S) => TTA (L)

atpA	1524	−	LSC	773	258	258	TCA (S) => TTA (L)
atpA	1524	−	LSC	791	264	264	CCC (P) => CTC (L)
atpB	1497	−	LSC	1184	395	395	TCA (S) => TTA (L)
atpF	555	−	LSC	92	31	31	CCA (P) => CTA (L)
atpI	744	−	LSC	349	117	120	CGG (R) => TGG (W)
atpI	744	−	LSC	629	210	213	TCA (S) => TTA (L)
ccsA	969	+	SSC	634	212	221	CAC (H) => TAC (Y)
clpP	609	−	LSC	224	75	75	ACA (T) => ATA (I)
clpP	609	−	LSC	503	168	168	GCT (A) => GTT (V)
clpP	609	−	LSC	559	187	187	CAT (H) => TAT (Y)
matK	1527	−	LSC	52	18	18	CTT (L) => TTT (F)
matK	1527	−	LSC	283	95	95	CTT (L) => TTT (F)
matK	1527	−	LSC	362	121	124	CCC (P) => CTC (L)
matK	1527	−	LSC	479	160	163	ACT (T) => ATT (I)

matK	1527	−	LSC	650	217	220	TCC (S) => TTC (F)
matK	1527	−	LSC	715	239	242	CCA (P) => TCA (S)
matK	1527	−	LSC	908	303	306	CCC (P) => CTC (L)
ndhA	1089	−	SSC	431	144	144	TCG (S) => TTG (L)
ndhA	1089	−	SSC	526	176	179	CCT (P) => TCT (S)
ndhA	1089	−	SSC	647	216	219	CCA (P) => CTA (L)
ndhA	1089	−	SSC	668	223	226	TCC (S) => TTC (F)
ndhA	1089	−	SSC	751	251	255	CAC (H) => TAC (Y)
ndhA	1089	−	SSC	812	271	275	CCC (P) => CTC (L)
ndhA	1089	−	SSC	964	322	326	CAC (H) => TAC (Y)
ndhA	1089	−	SSC	1031	344	348	CCC (P) => CTC (L)
ndhB	1533	+	IR	149	50	50	TCA (S) => TTA (L)

ndhB	1533	+	IR	467	156	156	CCA (P) => CTA (L)
ndhB	1533	+	IR	542	181	181	ACG (T) => ATG (M)
ndhB	1533	+	IR	586	196	196	CAT (H) => TAT (Y)
ndhB	1533	+	IR	611	204	204	TCA (S) => TTA (L)
ndhB	1533	+	IR	737	246	246	CCA (P) => CTA (L)
ndhB	1533	+	IR	746	249	249	TCT (S) => TTT (F)
ndhB	1533	+	IR	830	277	277	TCA (S) => TTA (L)
ndhB	1533	+	IR	836	279	279	TCA (S) => TTA (L)
ndhB	1533	+	IR	902	301	301	ACC (T) => ATC (I)
ndhB	1533	+	IR	1255	419	419	CAT (H) => TAT (Y)
ndhB	1533	+	IR	1481	494	494	CCA (P) => CTA (L)
ndhD	1503	−	SSC	146	49	49	ACA (T) => ATA (I)
ndhD	1503	−	SSC	668	223	224	CCA (P) => CTA (L)

ndhD	1503	–	SSC	985	329	331	CCC (P) => TCC (S)
ndhD	1503	–	SSC	1081	361	363	CCA (P) => TCA (S)
ndhD	1503	–	SSC	1150	384	386	CAT (H) => TAT (Y)
ndhD	1503	–	SSC	1343	448	453	ACG (T) => ATG (M)
ndhD	1503	–	SSC	1414	472	481	CTT (L) => TTT (F)
ndhF	2226	–	SSC	392	131	131	TCC (S) => TTC (F)
ndhF	2226	–	SSC	586	196	196	CTT (L) => TTT (F)
ndhF	2226	–	SSC	623	208	208	TCA (S) => TTA (L)
ndhF	2226	–	SSC	1385	462	462	TCT (S) => TTT (F)
ndhF	2226	–	SSC	1567	523	526	CTT (L) => TTT (F)
ndhF	2226	–	SSC	1691	564	568	ACT (T) => ATT (I)
ndhF	2226	–	SSC	1846	616	625	CTT (L) => TTT (F)
ndhF	2226	–	SSC	2177	726	735	TCA (S) => TTA (L)
ndhG	531	–	SSC	148	50	50	CAT (H) => TAT (Y)
ndhG	531	–	SSC	155	52	52	CCA (P) => CTA (L)

ndhG	531	−	SSC	166	56	56	CAT (H) => TAT (Y)
ndhG	531	−	SSC	314	105	105	ACA (T) => ATA (I)
petB	648	+	LSC	418	140	140	CGG (R) => TGG (W)
petB	648	+	LSC	611	204	204	CCA (P) => CTA (L)
petL	96	+	LSC	5	2	4	CCT (P) => CTT (L)
psbE	252	−	LSC	214	72	72	CCT (P) => TCT (S)
psbF	120	−	LSC	77	26	26	TCT (S) => TTT (F)
rpl2	825	+	IR	2	1	1	ACG (T) => ATG (M)
rpl20	354	−	LSC	235	79	79	CAT (H) => TAT (Y)
rpl20	354	−	LSC	257	86	86	TCG (S) => TTG (L)
rpl20	354	−	LSC	278	93	93	CCC (P) => CTC (L)
rpoA	1035	−	LSC	368	123	123	TCA (S) => TTA (L)
rpoA	1035	−	LSC	830	277	279	TCA (S) => TTA (L)

rpoA	1035	–	LSC	920	307	309	ACG (T) => ATG (M)
rpoB	3213	–	LSC	473	158	159	TCA (S) => TTA (L)
rpoB	3213	–	LSC	566	189	190	TCG (S) => TTG (L)
rpoB	3213	–	LSC	623	208	209	CCG (P) => CTG (L)
rpoB	3213	–	LSC	2426	809	827	TCA (S) => TTA (L)
rpoC1	2049	–	LSC	41	14	14	TCA (S) => TTA (L)
rpoC1	2049	–	LSC	1447	483	500	CGT (R) => TGT (C)
rpoC1	2049	–	LSC	1946	649	674	ACT (T) => ATT (I)
rpoC2	4158	–	LSC	1493	498	517	ACG (T) => ATG (M)
rpoC2	4158	–	LSC	1586	529	548	TCT (S) => TTT (F)
rpoC2	4158	–	LSC	1774	592	613	CGT (R) => TGT (C)
rpoC2	4158	–	LSC	2287	763	948	CGG (R) => TGG (W)
rpoC2	4158	–	LSC	2812	938	1131	CCT (P) => TCT (S)

rpoC2	4158	–	LSC	3722	1241	1453	TCA (S) => TTA (L)
rps2	711	–	LSC	233	78	83	CCC (P) => CTC (L)
rps2	711	–	LSC	310	104	109	CGG (R) => TGG (W)
rps2	711	–	LSC	443	148	153	CCT (P) => CTT (L)
rps2	711	–	LSC	631	211	218	CCA (P) => TCA (S)
rps2	711	–	LSC	644	215	222	ACC (T) => ATC (I)
rps8	399	–	LSC	182	61	61	TCA (S) => TTA (L)
rps14	303	–	LSC	149	50	53	CCA (P) => CTA (L)
rps16	267	–	LSC	143	48	48	TCA (S) => TTA (L)
rps16	267	–	LSC	212	71	71	TCA (S) => TTA (L)
ycf3	507	–	LSC	44	15	15	TCT (S) => TTT (F)
ycf3	507	–	LSC	407	136	136	TCC (S) => TTC (F)

Supplementary Table 10. The list of accession numbers of the chloroplast genome sequences was used in this study.

No.	Taxon	Family	GenBank
1	*Macleaya microcarpa*	Papaveraceae	MH394383.1
2	*Coreanomecon hylomeconoides*	Papaveraceae	KT274030.1
3	*Papaver somniferum*	Papaveraceae	KU204905.1
4	*Papaver rhoeas*	Papaveraceae	MF943221.1
5	*Papaver orientale*	Papaveraceae	MF943222.1
6	*Meconopsis racemosa*	Papaveraceae	MK533649
7	*Meconopsis racemosa*（MH394401）	Papaveraceae	MH394401
8	*Meconopsis integrifolia (Maxim.) Franch*	Papaveraceae	MK533647
9	*Meconopsis horridula*	Papaveraceae	MK533646
10	*Meconopsis punicea*	Papaveraceae	MK533648
11	*Decaisnea insignis*	Lardizabalaceae	KY200671.1
12	*Euptelea pleiosperma*	Eupteleaceae	KU204900.1
13	*Berberis koreana*	Berberidaceae	KM057375.1
14	*Berberis amurensis var. latifolia*	Berberidaceae	KM057377.1
15	*Berberis amurensis*	Berberidaceae	KM057374.1
16	*Berberis amurensis var. quelpaertensis*	Berberidaceae	KM057376.1
17	*Diphylleia sinensis*	Berberidaceae	MG593057.1
18	*Diphylleia cymosa*	Berberidaceae	MG593058.1
19	*Diphylleia grayi*	Berberidaceae	MG593049.1
20	*Sinopodophyllum hexandrum voucher SHEX20150512*	Berberidaceae	KR779994.1
21	*Sinopodophyllum hexandrum*	Berberidaceae	MG593048.1

22	*Plagiorhegma dubium*	Berberidaceae	MG397139.1
23	*Gymnospermium microrrhynchum*	Berberidaceae	KM057373.1
24	*Nandina domestica*	Berberidaceae	DQ923117.1
25	*Stephania japonica*	Menispermaceae	KU204903.1
26	*Clematis brevicaudata*	Ranunculaceae	MG675223.1
27	*Naravelia zeylanica*	Ranunculaceae	MG675224.1
28	*Clematis repens*	Ranunculaceae	MG675222.1
29	*Clematis alternata*	Ranunculaceae	MG675221.1
30	*Anemone henryi*	Ranunculaceae	MG001340.1
31	*Clematis fusca var. coreana voucher PG003*	Ranunculaceae	KM652489.1
32	*Clematis terniflora*	Ranunculaceae	KJ956785.1
33	*Naravelia pilulifera*	Ranunculaceae	KY120887.1
34	*Coptis chinensis*	Ranunculaceae	KY120323.1
35	*Coptis quinquesecta isolate WLHL*	Ranunculaceae	MG585353.1
36	*Clematis alternata isolate Tamura 1*	Ranunculaceae	MG573152.1
37	*Trollius chinensis*	Ranunculaceae	KX752098.1
38	*Urophysa rockii*	Ranunculaceae	MH006686.2
39	*Semiaquilegia adoxoides*	Ranunculaceae	MH142265.2
40	*Urophysa henryi*	Ranunculaceae	MH142266.2
41	*Sabia yunnanensis*	Sabiaceae	KU204902.1
42	*Nelumbo nucifera*	Nelumbonaceae	KF009944.1

Supplementary Fig.1 Phylogenetic tree reconstruction of the 13 species inferred from maximum likelihood (ML), based on *matK* genes.

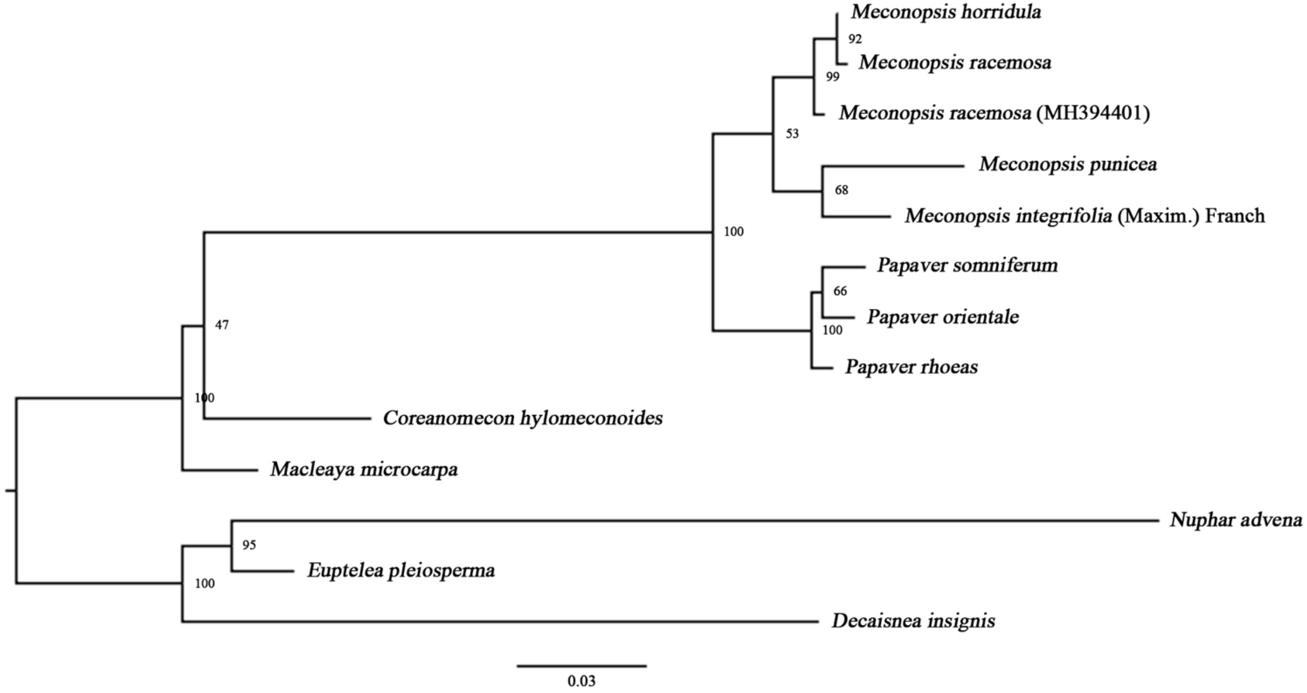

Supplementary Fig.2 Phylogenetic tree reconstruction of the 13 species inferred from maximum likelihood (ML), based on *ndhF* genes.

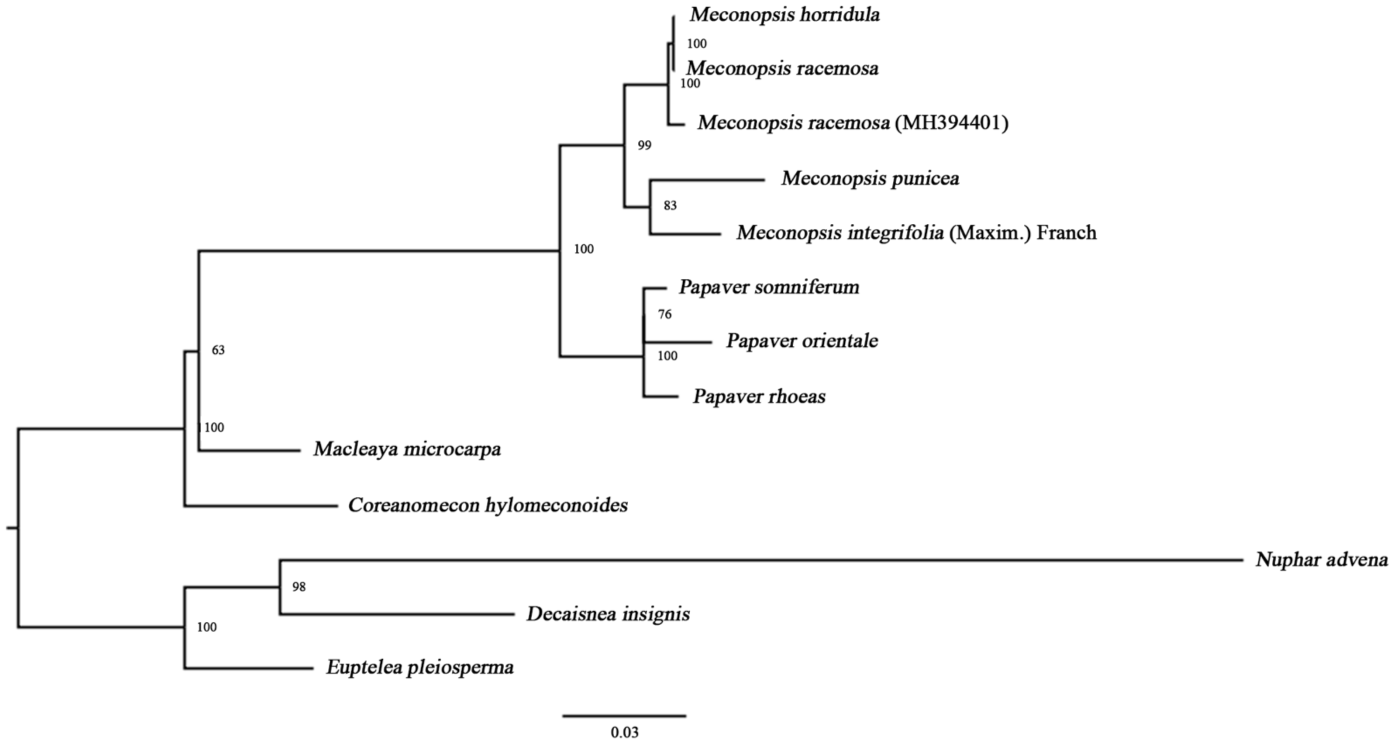

Supplementary Fig.3 Phylogenetic tree reconstruction of the 13 species inferred from maximum likelihood (ML), based on *petA* genes.

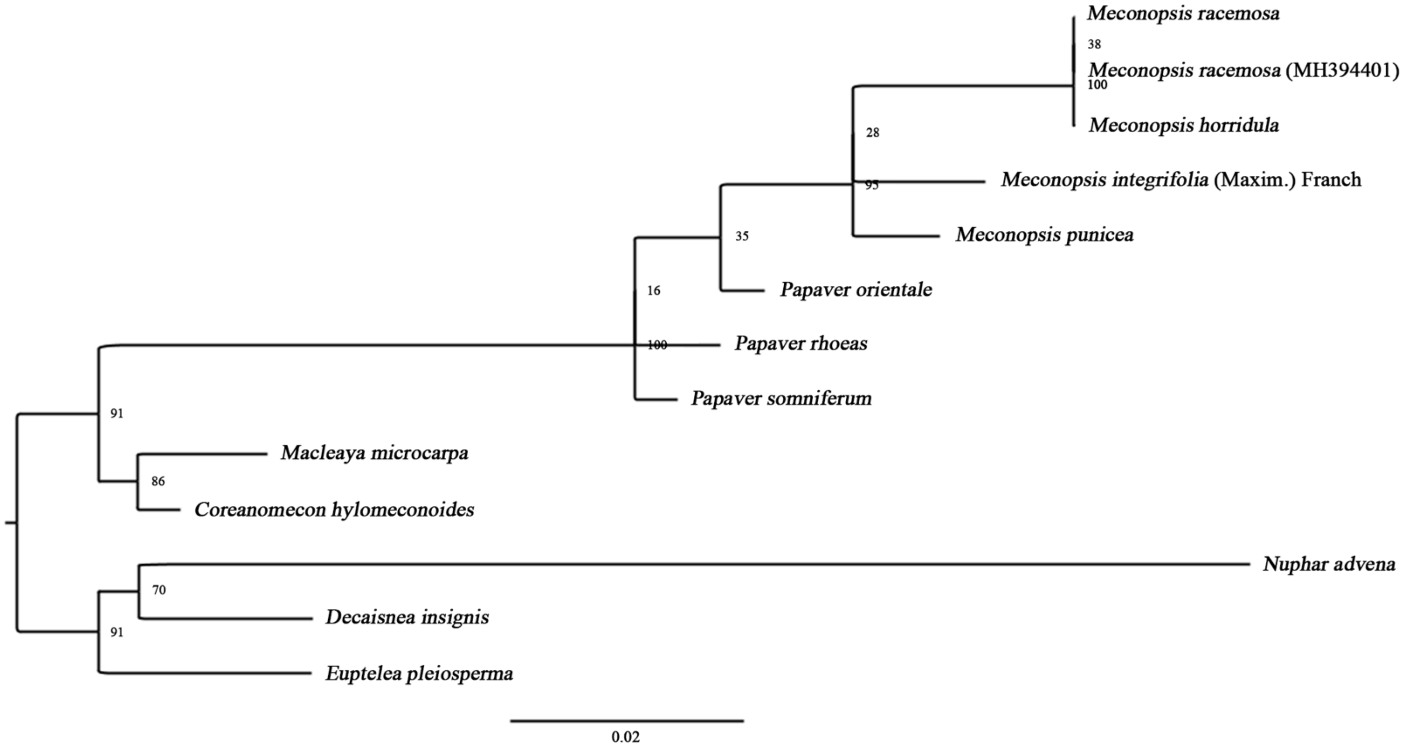

Supplementary Fig.4 Phylogenetic tree reconstruction of the 13 species inferred from maximum likelihood (ML), based on *rpoC2* genes.

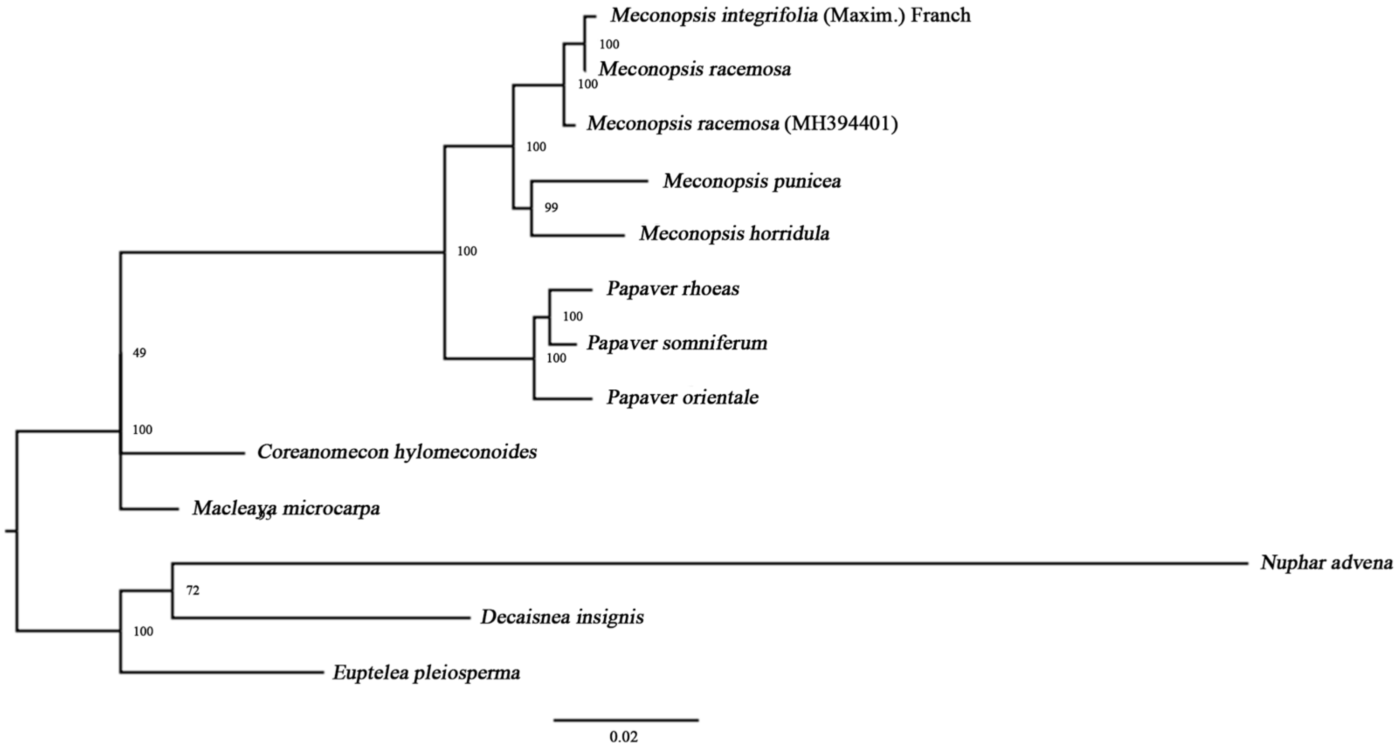

Supplement: Supplementary file 1 — Supplementary [file 41598_2019_47008_MOESM1_ESM.pdf]
